# Chromosome-level assembly of the *Phytophthora agathidicida* genome reveals adaptation in effector gene families

**DOI:** 10.3389/fmicb.2022.1038444

**Published:** 2022-11-02

**Authors:** Murray P. Cox, Yanan Guo, David J. Winter, Diya Sen, Nicholas C. Cauldron, Jason Shiller, Ellie L. Bradley, Austen R. Ganley, Monica L. Gerth, Randy F. Lacey, Rebecca L. McDougal, Preeti Panda, Nari M. Williams, Niklaus J. Grunwald, Carl H. Mesarich, Rosie E. Bradshaw

**Affiliations:** ^1^Laboratory of Molecular Plant Pathology/Bioprotection Aotearoa, School of Natural Sciences, Massey University, Palmerston North, New Zealand; ^2^Institute of Environmental Science and Research (ESR), Porirua, New Zealand; ^3^Scion, Rotorua, New Zealand; ^4^Department of Botany and Plant Pathology, Oregon State University, Corvallis, OR, United States; ^5^Laboratory of Molecular Plant Pathology/Bioprotection Aotearoa, School of Agriculture and Environment, Massey University, Palmerston North, New Zealand; ^6^School of Biological Sciences and Digital Life Institute, University of Auckland, Auckland, New Zealand; ^7^Bioprotection Aotearoa, School of Biological Sciences, Victoria University of Wellington, Wellington, New Zealand; ^8^Plant and Food Research, Lincoln, New Zealand; ^9^Plant and Food Research, Hawkes Bay, New Zealand; ^10^Horticultural Crops Disease and Pest Management Research Unit, USDA Agricultural Research Service, Corvallis, OR, United States

**Keywords:** *Phytophthora*, oomycete, kauri dieback, forest disease, chromosome-level genome assembly, chromatin conformation capture, effectors

## Abstract

*Phytophthora* species are notorious plant pathogens, with some causing devastating tree diseases that threaten the survival of their host species. One such example is *Phytophthora agathidicida*, the causal agent of kauri dieback – a root and trunk rot disease that kills the ancient, iconic and culturally significant tree species, *Agathis australis* (New Zealand kauri). A deeper understanding of how *Phytophthora* pathogens infect their hosts and cause disease is critical for the development of effective treatments. Such an understanding can be gained by interrogating pathogen genomes for effector genes, which are involved in virulence or pathogenicity. Although genome sequencing has become more affordable, the complete assembly of *Phytophthora* genomes has been problematic, particularly for those with a high abundance of repetitive sequences. Therefore, effector genes located in repetitive regions could be truncated or missed in a fragmented genome assembly. Using a combination of long-read PacBio sequences, chromatin conformation capture (Hi-C) and Illumina short reads, we assembled the *P. agathidicida* genome into ten complete chromosomes, with a genome size of 57 Mb including 34% repeats. This is the first *Phytophthora* genome assembled to chromosome level and it reveals a high level of syntenic conservation with the complete genome of *Peronospora effusa*, the only other completely assembled genome sequence of an oomycete. All *P. agathidicida* chromosomes have clearly defined centromeres and contain candidate effector genes such as RXLRs and CRNs, but in different proportions, reflecting the presence of gene family clusters. Candidate effector genes are predominantly found in gene-poor, repeat-rich regions of the genome, and in some cases showed a high degree of duplication. Analysis of candidate RXLR effector genes that occur in multicopy gene families indicated half of them were not expressed *in planta*. Candidate CRN effector gene families showed evidence of transposon-mediated recombination leading to new combinations of protein domains, both within and between chromosomes. Further analysis of this complete genome assembly will help inform new methods of disease control against *P. agathidicida* and other *Phytophthora* species, ultimately helping decipher how *Phytophthora* pathogens have evolved to shape their effector repertoires and how they might adapt in the future.

## Introduction

*Phytophthora* is an oomycete genus named for its reputation as a ‘plant destroyer’. Some species are notorious pathogens of food crops (Fones et al., 2020). For example, *P. infestans* has been known as a potato pathogen since the 19th century and *P. sojae* was identified as a soybean pathogen in the 1950s (Govers and Gijzen, 2006). Since 2000, however, the number of *Phytophthora* species identified as pathogens in forest ecosystems has increased around the world (Hansen, 2015). For example, *P. ramorum* was discovered as the causal agent of sudden oak death in the United States (Rizzo et al., 2002), *P. kernoviae* on beech in England (Brasier et al., 2005) and *P. pluvialis* on pines in New Zealand (Dick et al., 2014). More *Phytophthora* species are being discovered and identified as new disease outbreaks are investigated and new species identified (Hansen, 2015; Scott et al., 2019; Keriö et al., 2020).

*Phytophthora agathidicida*, which is in *Phytophthora* Clade 5, causes a dieback disease of New Zealand kauri trees known as kauri dieback (Weir et al., 2015). The pathogen invades and colonises the roots and vascular tissue, resulting in resin secretion (gummosis) from trunk lesions, crown decline and eventual death of the tree (Bradshaw et al., 2020). *P. agathidicida* is currently thought to have a narrow host range and limited geographic distribution (Bradshaw et al., 2020). While it is generally considered an introduced pathogen, its origin is unknown (Winkworth et al., 2021). The host of this disease, New Zealand kauri (*Agathis australis* (D.Don) Loudon), is part of the ancient Araucariaceae family, with trees capable of living for over 1,500 years (Ahmed and Ogden, 1987). Kauri is an iconic species with immense cultural significance for indigenous Māori people and is an ecologically vital foundation species for many of New Zealand’s native forests (Wyse et al., 2014; Black et al., 2018; Lambert et al., 2018).

An important focus of oomycete research has been the identification and analysis of proteinaceous effectors that are produced by the pathogens. These include intracellular effectors such as RXLR and CRN (crinkling and necrosis) proteins, and apoplastic effectors such as elicitins, NLPs (necrosis-like proteins) and some CAZymes (carbohydrate-active enzymes) that collectively facilitate pathogen invasion of their hosts (Amaro et al., 2017; Wang et al., 2019b; Rocafort et al., 2020; Chepsergon et al., 2021; Bradley et al., 2022; Wang et al., 2022; Wilson and McDowell, 2022). In some cases, plant hosts have evolved to recognise specific pathogen effectors by means of immune receptors, which then elicit plant defence responses. Pathogens, however, can evolve to avoid recognition through deletion, mutation or silencing of these effectors, provided that a significant associated fitness penalty is not incurred (Pais et al., 2018; Dong and Ma, 2021).

The availability of pathogen genome sequences has revolutionised many aspects of plant pathology. These sequences facilitate an unprecedented level of knowledge that can be exploited for many purposes such as developing diagnostic tools, screening for pathogen effectors to identify host resistance and making disease predictions based on patterns of pathogen dispersal and evolution (Hamelin and Roe, 2020; Keriö et al., 2020). Draft genome sequences are now available for many *Phytophthora* pathogens of forest trees (Tyler et al., 2006; Quinn et al., 2013; Feau et al., 2016; Grenville-Briggs et al., 2017; Vetukuri et al., 2018; McGowan et al., 2020; Thorpe et al., 2021), including an earlier draft genome of *P. agathidicida* (Studholme et al., 2016). However, this previously assembled *P. agathidicida* genome sequence, like those of many other *Phytophthora* species obtained using short-read Illumina sequencing, was highly fragmented with >3,000 scaffolds (Haas et al., 2009; Studholme et al., 2016; Fletcher and Michelmore, 2018; Guo et al., 2020a). The prevalence of repeated sequences makes *Phytophthora* genomes recalcitrant to assembly. Repetitive, transposon-rich regions of oomycete genomes are often enriched in effector genes, facilitating the adaptive evolution of those genes (Dong et al., 2015; Zhang et al., 2019). Thus, annotation of genome assemblies in which some repetitive regions are missing often excludes some effector genes. For instance, a high-quality genome assembly of *P. cinnamomi* led to the identification of candidate effector genes that were not present in an earlier, more fragmented, genome assembly (Engelbrecht et al., 2021).

The importance of high-quality genome assemblies for accurate and complete identification of candidate effector genes has led to considerable efforts to exploit long-read sequencing technologies to create better-assembled genome sequences (Fletcher and Michelmore, 2018). For example, scaffolds numbering in the thousands from short-read (principally Illumina) technologies have been reduced to hundreds for some species including *P. capsici* (Cui et al., 2019; Stajich et al., 2021), *P. cinnamomi* (Engelbrecht et al., 2021) and *P. sojae* (Fang et al., 2020), and less than 30 scaffolds for *P. ramorum* (Carleson et al., 2022). However, despite recent advances in long-read sequencing technologies and assembly techniques (Fletcher and Michelmore, 2018), it has proven difficult to achieve chromosome-level assemblies of *Phytophthora* genomes, principally due to their high repeat content, frequent heterozygosity and relatively large size.

Here we report the chromosome-level sequence assembly of the 57 Mb *P. agathidicida* genome and chromosome-level distributions of key classes of genes and structural genomic elements. This genome assembly will not only provide an important foundation for better understanding the molecular mechanisms underpinning virulence and pathogenicity by the kauri dieback pathogen, but will also act as a reference for other *Phytophthora* species.

## Materials and methods

### *Phytophthora agathidicida* isolate

The type strain of *Phytophthora agathidicida* B.S. Weir, Beever, Pennycook & Bellgard, isolate NZFS3770 (International Collection of Microorganisms [ICMP] 17,027), was initially collected in 2006 from Great Barrier Island (Aotea), New Zealand (WGS84 coordinates 175.417921E 36.225347S). The Ngāti Rehua Ngātiwai ki Aotea Trust has cultural authority over this isolate, which is hereafter referred to as *P. agathidicida* 3770.

### DNA extraction and sequencing

For genomic DNA (gDNA) extraction, *P. agathidicida* 3770 mycelium was grown on cellophane membranes on V8 agar plates (Weir et al., 2015) at 22°C in the dark for 5 days before harvesting and freeze-drying. High molecular weight genomic DNA was extracted using a Blood and Cell Culture DNA Midi Kit (Qiagen, Hilden, Germany) and a 100G Genomic-tip 100/G (Qiagen). Genomic DNA quality and quantity were assessed on a 0.4% agarose gel and by using a Qubit Fluorometer (Life Technologies, Singapore). For whole-genome sequencing, a *P. agathidicida* genomic DNA library (subread length 20–30 kb) was prepared and sequenced on a PacBio Sequel II platform by Novogene (Hong Kong), *via* the Massey University Genome Service, yielding 7.7 million reads, average length 19.9 kb, N50 = 32.7 kb. The same genomic DNA sample was also sequenced on an Illumina Novaseq™ 6000 platform, yielding 34 million 150 bp paired-end reads.

### Hi-C libraries and sequencing

For Hi-C analysis, *P. agathidicida* 3770 mycelium was grown in triplicate in V8 broth with shaking at 180 rpm for 4 days at 22°C in the dark. Mycelium was harvested from each replicate flask and divided such that half was used for the Hi-C library (pooled from three replicates) and half retained for RNA extraction (three separate replicates).

The Hi-C library was made using a Proximo Hi-C (Fungal) kit (Phase Genomics, Seattle, WA), following the manufacturer’s instructions. The quality and quantity of the Hi-C library were checked using a LabChip® GX Touch HT Nucleic Acid Analyser (Perkin Elmer, Melbourne, Australia), with the library then sequenced on an Illumina Novaseq™ 6000 platform, yielding 310 million 150 bp paired-end reads.

### RNA extraction and sequencing

For Iso-Seq analysis, RNA was extracted from *P. agathidicida* 3770 grown under a diverse range of growth conditions in order to maximise the number and diversity of genes represented; namely (1) mycelia grown in clarified V8, potato dextrose (PD) and plich broth (Meijer et al., 2014) at 22°C for 3 days without shaking; (2) mycelium incubated for 1.5, 3, 6 and 24 h in the presence of kauri leaves (from seeds originally sourced from Waipoua forest, New Zealand; Ye et al., 2011); and (3) a mixture of zoospores and cysts (Lawrence et al., 2017). RNA was extracted from the combined samples by using an RNeasy Plant Mini Kit (Qiagen). A cDNA Iso-Seq library with 1–10 kb reads was made by Novogene by pooling a size-selected library with a non-size-selected library to increase the diversity and yield of fragments >4 kb. This pooled library was sequenced on a PacBio Sequel (Novogene), yielding 34 million subreads with an average read length of 2.36 kb.

To complement the Hi-C data, RNA was also extracted from the *P. agathidicida* 3770 mycelium grown for Hi-C analysis (see above) using an RNeasy Plant Mini Kit (Qiagen). Gene expression was calculated from the three biological replicates, sequenced to a depth of over 30 million 150 bp paired-end reads per replicate, on the Illumina Novaseq™ 6000 platform. Reads mapping uniquely to single gene model exons were identified with STAR 2.7.10a (Dobin et al., 2013), using the gene annotations described below, and gene expression levels were subsequently classified by percentiles.

We took advantage of the new genome assembly and annotation described in this study to re-map pre-existing RNA-seq reads from another isolate of *P. agathidicida,* NZFS3813 (Herewini et al., 2018), that had been grown on kauri leaves and roots (from seeds originally sourced from Waipoua forest, NZ) in a project developed with the cultural authority of Te Roroa. The experimental design, growth conditions and RNA extraction were described previously (Guo et al., 2020a). RNA library construction and Illumina sequencing were performed by the Beijing Genomics Institute (BGI), as part of the Scion ‘Healthy Trees Healthy Future’ Programme, to yield 150 bp paired-end reads, with approximately 150 million reads from each of three replicates for 6 and 24 h samples, and 75 million reads for 48 and 72 h samples. We also prepared *in vitro* RNA samples for Pa3813 to enable comparison with *in planta* gene expression. For this, *P. agathidicida* 3813 was grown in V8 broth at 22°C for 5 days without shaking. RNA was extracted using a Spectrum™ Plant Total RNA Kit (Sigma) and sequenced with 150 bp paired-end reads by Novogene using an Illumina NovaSeq™ 6000.

The sequencing reads from all experimental conditions were mapped to the *P. agathidicida* 3770 genome using HISAT2 v2.2.1 (Kim et al., 2015, 2019), with default settings except without repeat-masking and with the addition of the ‘--no-unal’ setting to suppress the inclusion of unaligned reads in the output alignment file that are expected to derive from the kauri host. The default mapping parameters used in the Hisat2 mapping tool do not preclude multiple read mapping in the case of duplicated genes. Read counts were calculated for each gene using ‘featureCounts’ (Liao et al., 2014) in the R package Rsubread v2.10.5 (Liao et al., 2019). Differential gene expression between the *in vitro* samples and each of the other experimental conditions was performed with edgeR v3.38.4 (Robinson et al., 2010) using the quasi-likelihood pipeline (Chen et al., 2016). Gene expression data were transformed and expressed as log_2_ fragments per kilobase of exon per million mapped fragments (FPKM).

### Genome assembly and synteny analysis

Assemblies were produced from the PacBio data using three programs with different assembly algorithms, Canu v2.1 (Koren et al., 2017), Mecat2 v2020.02.28 (Xiao et al., 2017) and Flye v2.8.3 (Kolmogorov et al., 2019). The three algorithms complemented each other, with discontinuities present in the primary assembly from Canu often being well resolved in the others. The small number of non-assembled gaps could be resolved by identifying multiple reads with >10 kb of sequence matching each broken flank. As a conservative strategy, these gaps were closed by scaffolding the two contigs with a string of 10 Ns. The final assembly was polished with Pilon v1.23 (Walker et al., 2014) using 150 bp paired-end Illumina MiSeq reads produced as part of this project using the same gDNA sample as used for the PacBio libraries. Centromeres were identified by their characteristic cross-like patterns in the Hi-C contact map. Following standard practice, chromosomes were ordered by size, oriented with short arms first. The ribosomal DNA locus was manually annotated and, by convention, trimmed to two complete rDNA units together with the partial flanking units present at each terminus. Telomeres were trimmed to the last complete canonical repeat.

Large-scale synteny comparisons of the *P. agathidicida* 3770 genome relative to other oomycete species was determined by comparison to the 70-contig draft genome sequence of *P. sojae* isolate P6497 (NCBI accession number GCA_009848525.1; Fang et al., 2020), and to the genome of *Peronospora effusa* isolate UA202013 (GCA_021491655.1; the only other completely assembled genome sequence of an oomycete species; Fletcher et al., 2021). These comparisons were performed with D-GENIES v1.3.1 (Cabanettes and Klopp, 2018), using a cross-mapping algorithm that employs minimap2 v2.24-r1122 (Li, 2018).

### Repetitive elements analysis

We produced a *de novo* annotation of repeats in the *P. agathidicida* 3770 genome using the RepeatModeler v2.0.1 pipeline (Flynn et al., 2020). This pipeline uses RepeatScout to discover multicopy regions of the genome and RECON (Bao and Eddy, 2002) to discover known transposable element (TE) motifs. We used RepeatMasker v4.0.6 (Chen, 2004) to locate specific elements of each identified repetitive element family within the genome. Having already identified centromere locations with the Hi-C 3D data, we used BLASTn v2.5.0 to search the genome for elements with homology to the CoLT retrotransposons that occur in *P. sojae* centromeric regions (Fang et al., 2020). Genomic locations with more than 10 copies of a CoLT retrotransposon element confirmed the positions of centromeres in the *P. agathidicda* genome.

### Gene prediction and functional annotation

Genes encoding tRNAs were identified using tRNAscan-SE v2.0 (Lowe and Eddy, 1997) and non-protein-coding RNAs by searching v14.5 of the Rfam database (Kalvari et al., 2021) using the cmscan option from Infernal v1.1.2 (Nawrocki and Eddy, 2013) for all other RNA classes. We identified putative mRNA transcripts from our IsoSeq data using v3.0 of the PacBio IsoSeq pipeline^1^.

Protein-coding genes were identified from the masked *P. agathidicida* 3770 genome using Augustus v3.3.3 (Stanke et al., 2006). We generated training data for Augustus gene-calling using our IsoSeq transcripts following a protocol originally designed for expressed sequence tags (Hoff and Stanke, 2019), then used minimap2 v2.17 to align transcripts to the reference genome and the PASA v2.4.1 pipeline to curate the resulting alignments. Proteins from the current version of the *P. sojae* P6497 genome (Tyler et al., 2006) were also used as evidence in our gene calling pipeline.

To assess the completeness of the *P. agathidicida* 3770 genome assembly, BUSCO v4.14 (Benchmarking Universal Single-Copy Orthologs; Seppey et al., 2019) was used in genome mode with the stramenopile lineage and Eukaryota gene sets from OrthoDB (Zdobnov et al., 2021), using default parameter values. The BUSCO values were compared with those of other oomycete genome assemblies produced from long-read sequences, including those of *Pe. effusa*, (Fletcher et al., 2021) *P. ramorum* (Carleson et al., 2022) and *P. sojae* (Fang et al., 2020). The accuracy of our automated gene-calling was assessed by comparing the resulting gene models to hand-curated models produced from other studies focusing on RXLR effectors (Guo et al., 2020a) and carbohydrate metabolism (Bradley, 2022).

Functional domains in predicted proteins were identified using InterProScan v5.53. Signal peptides were predicted using SignalP v3.0 (Bendtsen et al., 2004) and transmembrane domain prediction was done using TMHMM v2.0 (Krogh et al., 2001). SignalP v3.0 was chosen as it was previously deemed to be the most sensitive version for the prediction of signal peptides in oomycete effector proteins (Sperschneider et al., 2015).

A set of six gene classes were selected for further investigation based on their known roles in virulence in other *Phytophthora* species. Candidate CRNs and RXLRs were annotated in both the predicted proteins and all 6-frame translations of the genomes from getorf (−find 1 -minsize 210) using EMBOSS v6.6.0.0. ORFs in intergenic spaces between predicted genes that encode candidate effectors were supplemented into the annotations using a custom Python script^2^.

Putative RXLR effector proteins were identified using previously described approaches (McGowan and Fitzpatrick, 2017; Guo et al., 2020a). Briefly, sequences were tested using three prediction methods (Bhattacharjee et al., 2006; Whisson et al., 2007; Win et al., 2007) implemented in a Python script (Cock et al., 2013). The intersection of candidates returned by all methods were considered RXLRs.

Sequences were putatively called as CRNs if they were identified from either a regular expression or a machine-learning approach. The regular expression approach implemented in effectR v1.0.2 (Tabima and Grünwald, 2019) was used to search for any sequence containing the LFLAK motif within or immediately following the first 90 amino acids. For the machine learning approach, an HMM profile was created from all 315 CRNs from *P. infestans* (Haas et al., 2009) aligned with MAFFT v7.480 (--legacygappenalty --genafpair --maxiterate 1000; Katoh and Standley, 2013) and hmmsearch v3.1b2 was used to identify sequences with a positive bit score. Lastly, any candidate not containing a degenerate LFLAK motif (L[FY]LA[RK]) within or immediately following the first 90 amino acids was removed (McGowan and Fitzpatrick, 2017; Thorpe et al., 2021).

Proteins were considered CAZymes if they were predicted as such by at least two of the three tools used by dbCAN2 (released 21 Dec 2021; HMMER, DIAMOND, and eCAMI; Zhang et al., 2018). Candidate elicitins and NLPs were identified on the basis of their pfam domains: PF00964 and PF05630, respectively (Derevnina et al., 2016). G-protein-coupled receptors (GPCRs) were predicted using the GPCR-Pen pipeline (Begum et al., 2020).

### Intergenic distance and 3D clustering analysis of gene classes

Intergenic 5′ and 3′ distances to the nearest gene (‘flanking intergenic regions’ or FIRs) were calculated for all genes, to determine the background distribution, as well as for the six gene classes of interest (CAZymes, CRNs, elicitins, GPCRs, NLPs and RXLRs). Custom code^3^ was used to plot the 5′ and 3′ distances against the background distribution. Monte Carlo probabilities that the median distance of each subset of genes is greater than that expected for the same number of randomly chosen genes was calculated *via* bootstrapping with replacement and corrected for multiple hypothesis testing using the false discovery rate approach. Individual genes were overlaid with the percentile of their expression level in culture.

Clustering of genes in the 3D space of the nucleus can be ascertained from Hi-C data. 3D interactions were calculated for the same six gene classes as above. Custom code^4^ calculated the mean number of pairwise Hi-C contacts between all genes in each class, and determined using Monte Carlo statistics whether their 3D interactions differed significantly from equivalently sized sets of genes chosen randomly across the genome. The Hi-C contact matrix had a window resolution of 10 kb. Probability values were corrected for multiple testing using the False Discovery Rate (FDR) implementation in the p.adjust function of R v4.2.0 (R Core Team, 2022).

### Single nucleotide variants

Illumina genome data were available for 13 additional New Zealand isolates of *P. agathidicida* as NCBI BioProjects PRJNA290658, PRJNA486676 and PRJNA864064 (Studholme et al., 2016; Guo et al., 2020a). These Illumina reads were aligned to the *P. agathidicida* 3770 reference genome using BWA-mem v0.7.17-r1188 (Li, 2013). Duplicate reads were marked by Picard Tools MarkDuplicates v2.21.8.^5^ Variants were called using GATK v4.1.8.1 (Van der Auwera and O'Connor, 2020). First, all samples were separately called with HaplotypeCaller (Poplin et al., 2018) (setting the -ERC parameter of GVCF to produce per sample genotype/gVCF files). Next, gVCF files were merged with GenomicsDBImport and finally joint-genotyped with GenotypeGVCF. Variants were filtered to retain single nucleotide polymorphisms (SNPs) using GATK SelectVariants. Next, VCFTools was used for retaining only biallelic sites (--min-alleles 2 --max-alleles 2) and hard filters were applied for missing data (max-missing = 0.8), minimum depth (minDP = 10) and minimum quality (minQ = 30). The final vcf file containing filtered variants from all the genomes was used for downstream analyses.

To estimate mean SNPs per exon, all exons were extracted from the *P. agathidicida* 3770 GFF genome annotation file into a BED file. Locations of SNPs from the joint-called VCF file containing SNPs from all isolates were also extracted into a BED file. SNPs overlapping exons were counted by using bedtools intersect with the two BED files followed by the -c option to count the number of overlaps. The results were summarised as means using the summarise function in the dplyr package (Wickham et al., 2015) in R v3.6.2.

### Ploidy and heterozygosity estimation and copy number variation analysis

For estimation of ploidy and heterozygosity, K-mer analysis was performed with *P. agathidicida* 3770 Illumina reads using the KMC algorithm (Deorowicz et al., 2013), then genome profiling was carried out with Genomescope v2.0 (Ranallo-Benavidez et al., 2020) using a K-mer size of 21.

Copy number variation (CNV) was analysed using CNVPytor (Suvakov et al., 2021), with the *P. agathidicida* 3770 assembly file used as the reference, followed by GC correction. BAM and VCF files for all the *P. agathidicida* isolate samples (outlined in Section “Single nucleotide variants”) were processed to extract read depth and allele frequency information using three different bin sizes (1, 5, and 10 kb) following protocols outlined in the manual.^6^ CNVs were called using the CNVpytor mean-shift method for all three bin sizes, and the results were compared manually. The final file contained copy number calls from the 5 kb bin size, with the column ‘CNV_level’ in the output file normalized to a copy number of 2. CNVs were visualised in KaryoploteR v3.15 (Gel and Serra, 2017) with the kpPlotRegions function and labelled as deletions (copy number < 2) or duplications (copy number > 2). Code for recreating the karyoploteR output is available online (https://github.com/diyasen2021/copynumber.R).

## Results

### A chromosome-level genome assembly of *Phytophthora agathidicida* 3770

#### The genome assembly

**Figure 1:**
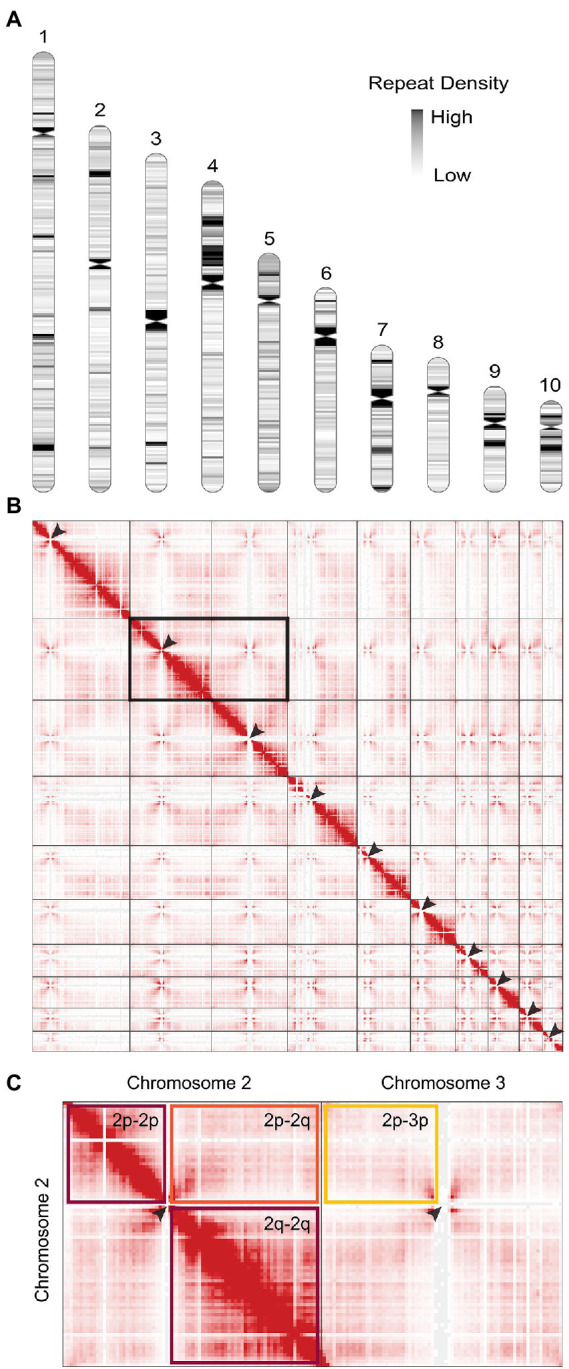
The genome of *Phytophthora agathidicida*. **(A)** Karyotype of the *P. agathidicida* 3770 genome, indicating centromere locations (pinched-in regions) and density of repeat elements across the ten complete chromosomes, although the full rDNA array is not depicted in chromosome 10. **(B)** Contact map of 3D DNA interactions within the *P. agathidicida* nucleus (red stippling). Arrows mark Hi-C patterns characteristic of centromere locations. **(C)** Enlarged example region of the DNA contact map shown in B. DNA interactions are frequent within the arms of each chromosome (maroon boxes), whilst interactions between chromosome arms are much reduced (orange box) and similar to interaction levels between different chromosomes (yellow box).

The previous genome assembly of *P. agathidicida* 3770 (Studholme et al., 2016; BioProject PRJNA290659) had 3,689 scaffolds and an estimated genome size of 37,328,500 bp. Here, we assembled the genome sequence into ten complete chromosomes with a genome size of 56,996,300 bp and a read depth of >100x (Figure 1A). To produce this chromosome-level genome assembly, PacBio reads were assembled with a combination of complementary algorithms to yield four whole chromosomes (telomere to telomere) and 14 chromosome fragments with one or no telomeres. Hi-C (chromatin conformation capture) reads and inspection of multiple long reads containing sequences from the ends of these fragments were then used to assemble the 14 chromosome fragments into six more chromosomes. Finally, Illumina reads were used to polish the sequence.

BUSCO analysis of the *P. agathidicida* genome assembly was 100% (complete and single copy genes) based on the Stramenopile lineage gene sets, indicating high accuracy of the assembly. This was a higher score than those for other oomycete genome assemblies based on long-read sequences, including those for *Pe. effusa*, *P. ramorum* and *P. sojae* (Supplementary Figure S1). Similarly, using the Eukaryota gene set, the complete and single copy BUSCO score for the *P. agathidicida* genome was higher than those for *Pe. effusa* and *P. sojae*, but similar to those of *P. ramorum* (Supplementary Figure S1). The number of predicted gene models increased from 17,691 (fragmented assembly from short-read sequencing data) to 17,963 (chromosome-level assembly). A k-mer analysis with Genomescope (Supplementary Figure S2) suggests that *P. agathidicida* 3770 is diploid, with an estimated low heterozygosity level of 0.02%.

Positions of the centromeres on the ten chromosomes were indicated by the frequency of Hi-C interactions (Figures 1B,C). Only chromosome 3 is metacentric, 2 and 4 are submetacentric, whilst the others are acrocentric. The ten chromosomes vary in length from 2.2 Mb to 10.5 Mb with 52.9–53.6% GC content (Table 1). Chromosome 10 showed the highest repeat density (51.4%) and lowest gene density (Table 1), and chromosome 4 showed large blocks of repetitive sequences (Figure 1A) despite having a percentage repeats value similar to the genome mean. The ribosomal RNA gene array (rDNA) is at a single location on chromosome 10 and is estimated to contain 245 copies of the rDNA units (Supplementary Data S1), although the final reference assembly only includes two full repeats plus the two partial flanking units as outlined in the methods. Including the full 245 rDNA copies, chromosome 10 is actually ~4.7 Mb in length and the whole genome ~59.5 Mb. The mitochondrial sequence assembly was published previously (Winkworth et al., 2021; BioProject PRJNA738084).

**Table 1:** *Phytophthora agathidicida* genome features by chromosome.

Chromosome	Length bp	Protein-coding genes	tRNA genes	Genes /Mb	% GC	% Repeats
1	10,534,528	3,058	229	312	52.9	33.3
2	8,780,368	2,755	132	329	52.9	27.8
3	8,108,413	2,406	548	364	53.5	28.4
4	7,450,011	2,071	269	314	53.0	36.4
5	5,717,954	1,491	257	306	53.0	38.4
6	4,894,808	1,304	113	289	53.2	34.2
7	3,521,682	834	112	267	53.2	46.3
8	3,225,653	926	78	311	53.8	28.7
9	2,539,455	749	24	304	53.6	40.8
10	2,186,599	423	120	248	53.2	51.4
Mitochondrion	36,829	39	25	733	21.6	7.9
Total/Mean	56,996,300	16,056	1,907	314	53.1	34.3

#### The assembled genome shows synteny to other assembled oomycete genomes

**Figure 2:**
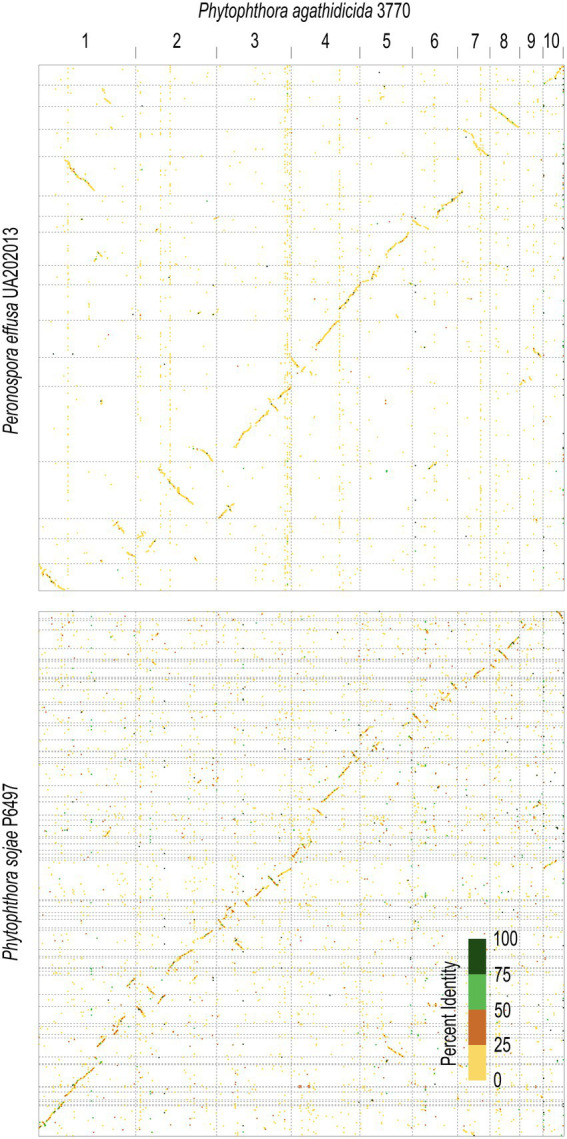
Comparative genomics of the *Phytophthora agathidicida* genome. Synteny was determined between the ten chromosomes of *P. agathidicida* 3770 and the complete genome of *Peronospora effusa* UA202013 (top panel) and the fragmentary genome assembly of *P. sojae* P6497 (bottom panel). Matching colors indicate percent sequence identity.

Comparative synteny analysis indicates substantial conservation of genome content between *P. agathidicida*, *P. sojae* and *Peronospora effusa*, with relatively little genome content unique to the three distinct species (Figure 2). Most of the conserved regions fall within long genomic blocks. Within the two completely assembled genome sequences, *P. agathidicida* chromosomes 7, 8 and 10 have essentially identical synteny with *Pe. effusa* across their entire length, despite being phylogenetically distant. This is suggestive of deep conservation of high-level genome organization, even though the DNA sequences themselves are divergent, as illustrated by low sequence identity (yellow and orange in the Figure 2 comparison plots). The comparative analysis also identifies species-specific genome rearrangements, such as an approximately 2 Mb inversion on *P. agathidicida* chromosome 3. Because the alternative non-inverted genomic structure is present in both *P. sojae* and *Pe. effusa*, the most parsimonious explanation is that this inversion occurred within the *P. agathidicida* lineage, with the ancestral state retained by the other two species. Given this high level of syntenic conservation, the chromosome-level genome of *P. agathidicida* may help guide potential scaffolding of more fragmented genome assemblies, as in this case for *P. sojae*.

#### Conserved centromere sequences and many unclassified repeats are present

**Figure 3:**
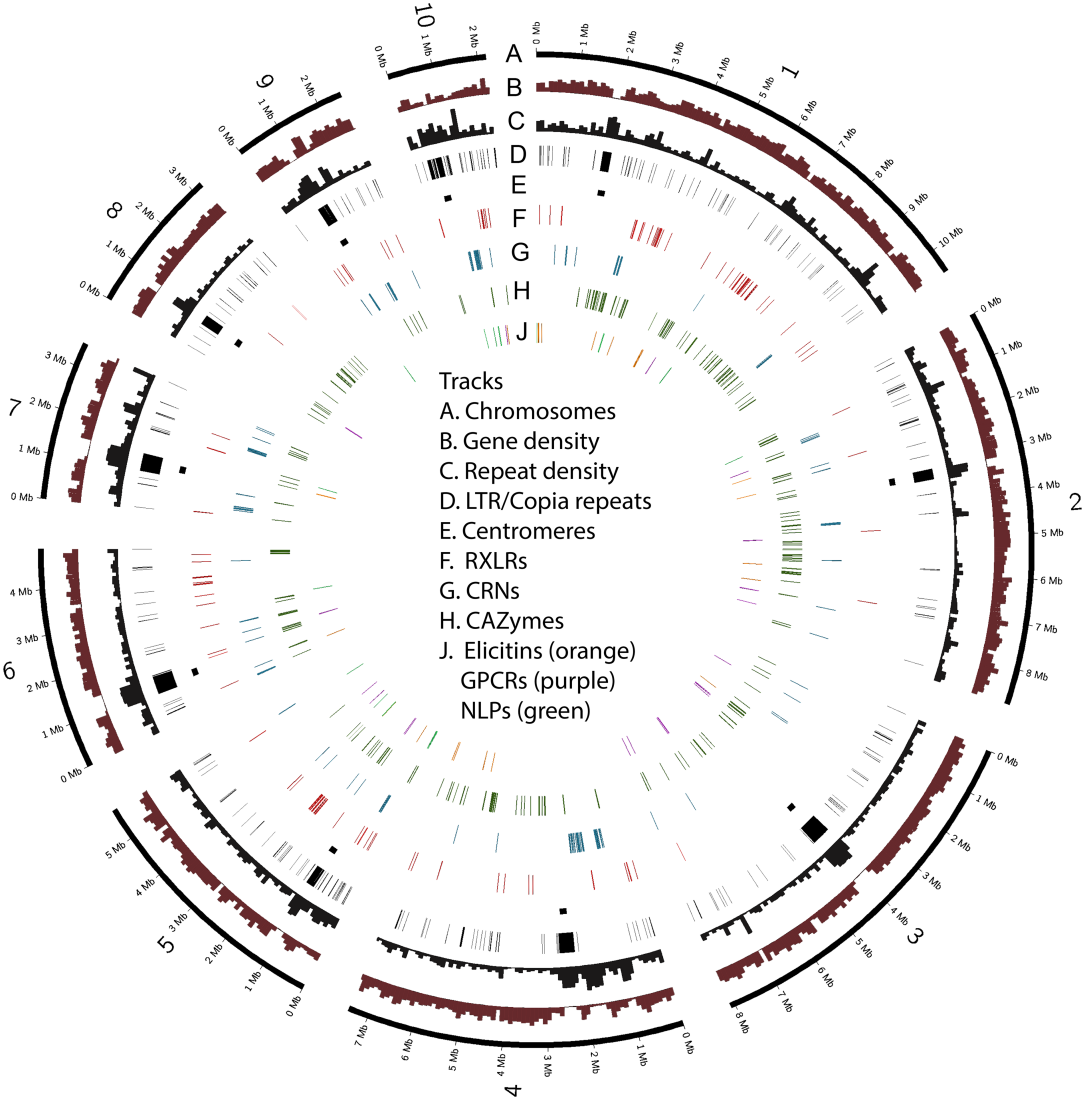
Chromosomes of *Phytophthora agathidicida* 3770. The circos plot illustrates the ten assembled chromosomes (numbered 1–10 on track A), along with densities of protein-coding genes and repetitive sequence (B, C); the positions of LTR/Copia (CoLT) repeats associated with centromeres (D, E) and genes in the six selected classes encoding predicted RXLR effectors, crinkling and necrosis proteins (CRNs), carbohydrate-active enzymes (CAZymes), elicitins, G-protein-coupled receptors (GPCRs) and necrosis-like proteins (NLPs) (F–J).

Over a third of the *P. agathidicida* 3770 genome was identified as being comprised of repeat elements (Table 1). A *de novo* annotation of repeats in the genome (Supplementary Table S1; Supplementary Figure S3) showed that the most common repeats are Long Terminal Repeat (LTR) retrotransposons, in particular LTR/Copia (CoLT) retrotransposons (~7% of the genome). The locations of particularly long blocks of CoLT elements coincide with centromeric regions identified by Hi-C analysis (Figure 3); the same type of retroelement was also found in centromeres of *P. sojae* (Fang et al., 2020). In the *P. agathidicida* genome, there are many more classes of DNA repeat elements (21) than other characterised repeat classes such as LTRs and LINEs (12), so whilst the contribution of each type of DNA element is small (at most 2% of the genome), they are cumulatively substantial (Supplementary Figure S3). There was low sequence diversity within some classes of repeats, suggesting recent transposon activity in *P. agathidicida* (Supplementary Figure S4). However, a large proportion of the repeats could not be classified, suggesting that there might be many repeats unique to the *Phytophthora* genus.

### Gene distributions

#### Some key gene groups show a non-uniform distribution across the genome

Candidate genes from six classes that have been characterised as virulence factors in other *Phytophthora* species (Hua et al., 2013; Ma et al., 2015; Amaro et al., 2017; Chepsergon et al., 2020; Pirc et al., 2022) were selected for further investigation. These are genes encoding candidate cytoplasmic RXLR and CRN effectors, apoplastic effectors including elicitins, NLPs, CAZymes, and GPCRs involved in signaling (Supplementary Table S2). Compared to the previous fragmented genome assembly in which 78 candidate RXLR genes were identified, 147 RXLR genes were called in the new genome assembly, even though these RXLR candidates were identified using intersection of the same three prediction methods (Bhattacharjee et al., 2006; Whisson et al., 2007; Win et al., 2007). However, in the new genome assembly, ORFs as well as annotated gene models were searched for RXLRs, thus this modified method plus the more complete genome assembly account for the increased RXLR gene count.

The number of RXLR gene candidates (147) was similar to those found in some other *Phytophthora* species, such as *P. chlamydospora* (132; McGowan et al., 2020) and *P. cactorum* (135; Yang et al., 2018), but less than in many other species including *P. ramorum* (400; Carleson et al., 2022) and *P. sojae* (350; Tyler et al., 2006), although we had taken a conservative approach to RXLR identification by excluding RXLR-like genes from our numbers. In contrast, the *P. agathidicida* genome contained more CRN gene candidates (145) than most sequenced *Phytophthora* genomes except *P. infestans* (196; Haas et al., 2009). For example, in two well-assembled *P. ramorum* genomes, only 45 and 47 CRN gene candidates were identified using the same methodology as we used for *P. agathidicida* (Carleson et al., 2022).

Some of the gene classes showed an asymmetric distribution across chromosomes (Table 2; Figure 3). For example, 60% of the candidate RXLR genes and 64% of the candidate NLP genes were found on chromosomes 1 or 5, whilst chromosomes 3 and 8 had no NLP genes and very few candidate RXLR, CRN and elicitin genes. For chromosome 10, despite its smaller size (2.2 Mb), lower gene density and higher repeat density compared to the other chromosomes (Table 1), there were representatives from all the studied gene groups and also a higher proportion of SNPs among a set of New Zealand *P. agathidicida* isolates, particularly in exons (Table 2).

**Table 2:** *Phytophthora agathidicida* candidate gene groups and single nucleotide polymorphisms (SNPs) by chromosome.

Chromosome	Genes encoding secreted proteins^a^	RXLRs	CRNs	Elicitins	NLPs	CAZymes	GPCRs	Mean SNPs^b^ /exon	Mean SNPs^b^ /kb
1	612	58	20	13	7	110	1	0.141	0.333
2	462	4	22	11	1	63	6	0.128	0.325
3	411	1	6	1	0	31	11	0.162	0.351
4	386	13	28	8	0	52	0	0.148	0.340
5	341	30	11	4	9	18	4	0.166	0.341
6	233	15	10	1	1	42	1	0.155	0.344
7	142	5	20	9	1	11	0	0.167	0.423
8	164	3	0	0	0	27	2	0.107	0.362
9	146	8	11	0	2	6	0	0.111	0.419
10	105	10	17	1	4	6	1	0.270	0.406
Total/Mean	3,002	147	145	48	25	366	26	0.147	0.364

a Proteins with a signal peptide predicted by SignalP v3.0.

b SNPs in the genome among 13 other isolates of *P. agathidicida* (Guo et al., 2020a) compared to *P. agathidicida* 3770.

**Figure 4:**
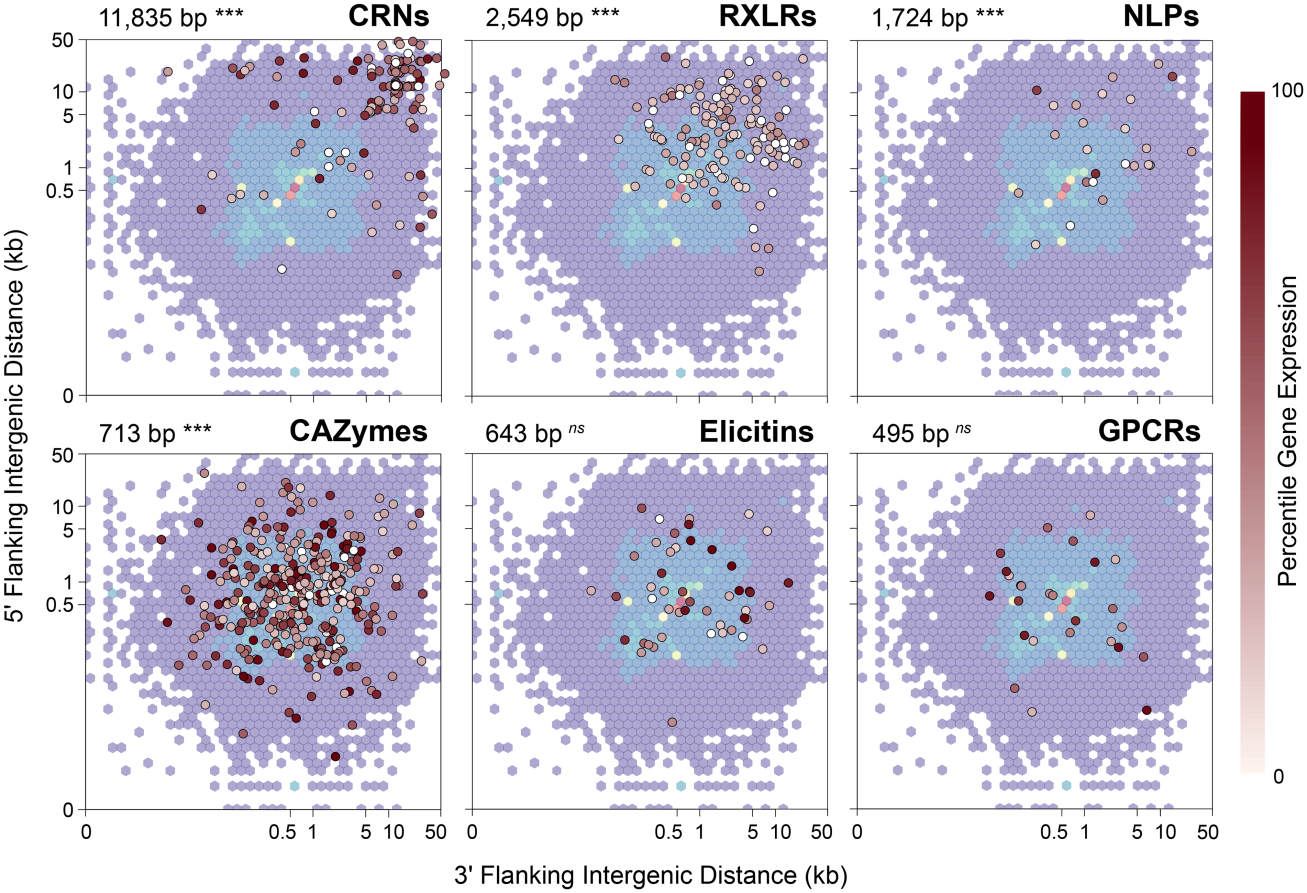
5′ and 3′ distances to the nearest gene in the *Phytophthora agathidicida* genome. 5′ and 3′ distances for all genes identified in the *P. agathidicida* 3770 genome are shown by the background density distribution, which is overlaid in each panel with intergenic distances for genes in six gene classes: RXLRs, CRNs, NLPs, CAZymes, elicitins and GPCRs. Points for individual genes are colored by their percentile expression level in culture, with no expression indicated in white, low expression by lighter reds and high expression by darker reds. The median intergenic distance and its statistical significance are also shown for each gene class. ***p < 0.001; ns, not significant.

A characteristic often noted in other filamentous plant pathogens, including *Phytophthora* species, is a compartmentalized genome in which genes involved in virulence or pathogenicity tend to be located in gene-sparse genomic regions enriched with repeats or transposable elements (Dong et al., 2015). As a consequence, effector genes tend to have longer intergenic distances to their neighboring genes when compared to other types of genes. To test whether *P. agathidicida* 3770 has this kind of compartmentalized genome, 5′ and 3′ distances to the nearest gene were calculated for genes in the six classes of interest: CAZymes, CRNs, elicitins, GPCRs, NLPs and RXLRs (Figure 4). The median 5′ and 3′ distance to the nearest gene is 512 bp for all genes identified in *P. agathidicida* 3770. Four gene classes show significantly larger median distances than this background rate: CRNs (11,835 bp), RXLRs (2,549 bp), NLPs (1,724 bp) and CAZymes (713 bp; all *p* < 1 × 10^−4^). Distances do not differ significantly for elicitins (643 bp, *p* = 0.74) or GPCRs (495 bp, *p* = 0.45).

We considered that distances to flanking genes might influence gene expression. RNA extracted from the same *P. agathidicida* samples as used for Hi-C analysis was analysed by RNA sequencing and genes then classified by expression level percentile. However, no association was observed visually between intergenic distances and percentile levels of gene expression (Figure 4).

#### 3D Genome clustering within gene classes reflects 2D arrangements on chromosomes

All six gene classes (CAZymes, CRNs, elicitins, GPCRs, NLPs and RXLRs) have 3D interaction profiles that differ significantly from gene sets chosen randomly from across the genome. CAZymes, elicitins, GPCRs, NLPs and RXLRs have larger numbers of 3D contacts (3- to 34-fold increase over a random gene set, all adjusted *p* < 0.001), indicating that on average these genes cluster more than average in the nucleus. Conversely, CRN genes exhibit fewer contacts (25-fold decrease) and therefore are less clustered in 3D space than average (adj *p* = 0.0006). As noted earlier, CRNs have markedly elevated 5′ and 3′ distances to the nearest gene along the chromosome (11,835 bp vs. 512 bp for all genes), reflecting their typical locations in repeat-rich and gene-poor regions of the genome. This physical isolation of CRN genes is probably associated with their greater isolation in 3D space.

Closer examination of genes in these six classes identified that many are physically adjacent along the genome, likely indicating that they arose through gene duplication, possibly followed by neofunctionalization. Genes that sit adjacent on a chromosome are by definition also close in 3D space, so we considered that the observed 3D clustering might simply be an artifact of the chromosomal co-location of many of these genes. To control for this feature, we excluded all genes that lay within 10 kb of any other gene in the same gene class and re-ran the analysis. After controlling for gene adjacency, the 3D clustering pattern disappears for elicitins, NLPs and RXLRs (all adj *p* > 0.05), but still holds, although more weakly, for GPCRs (6-fold increase in contacts over expectations, adj *p* = 0.036) and CAZymes (0.6-fold increase in contacts, adj *p* = 0.0002). The reduced 3D interaction of CRN genes is still strongly supported (21-fold decrease in contacts, adj p = 0.0006).

### A complete genome offers the potential for deeper insights into gene expression and adaptation

As well as providing a chromosome-level perspective of the genes and other genome features, a complete genome assembly based on a high coverage with very long read sequences provides greater confidence in gene organisation in regions where there are tandem repeats. We present here several examples to illustrate the possibilities for further analysis of a fully assembled genome.

#### Visualising genetic diversity in copy number variants across the genome

**Figure 5:**
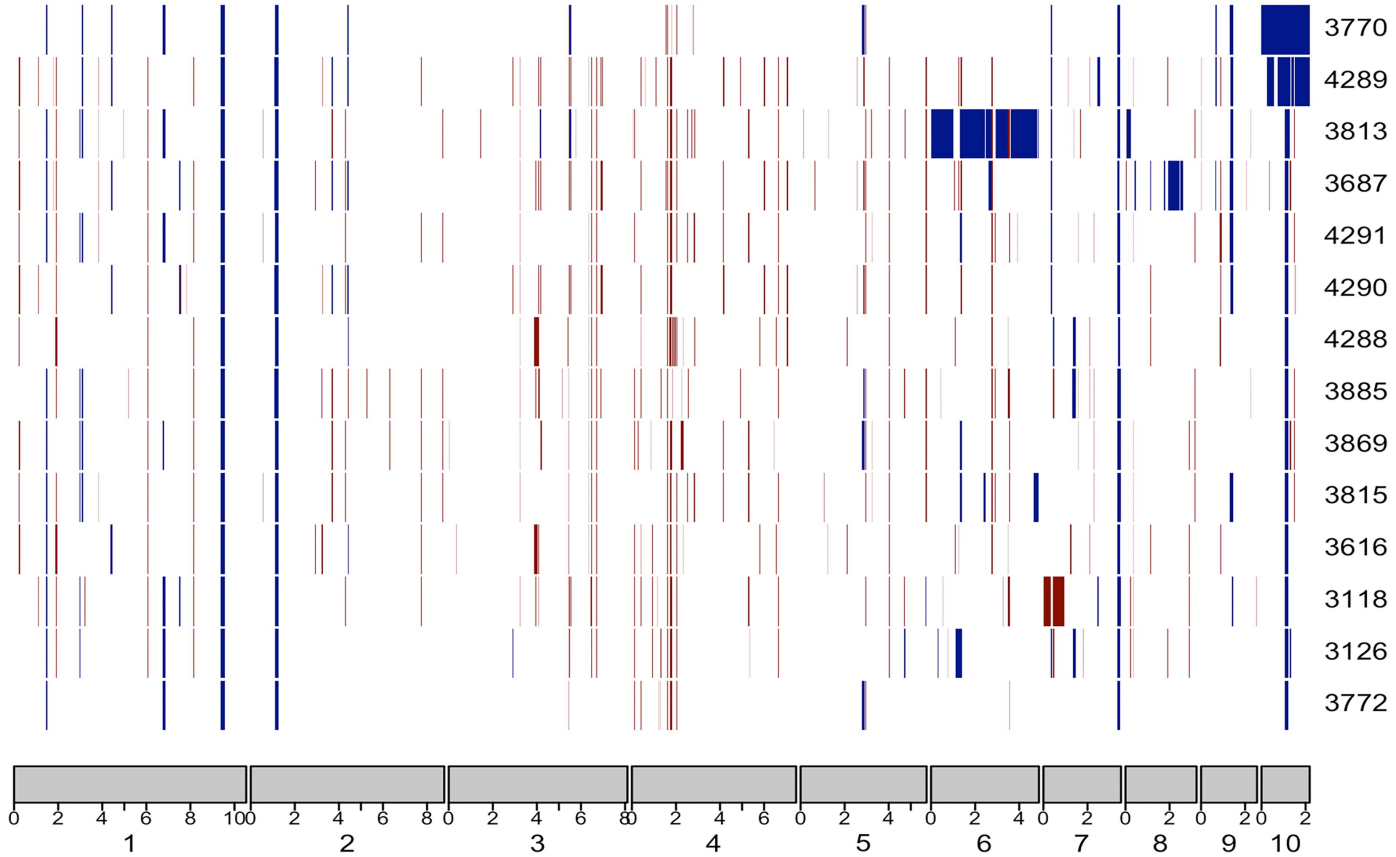
Copy number variants (CNVs) across the genomes of 14 isolates of *P. agathidicida* based on short read sequencing data. The rectangles at the bottom represent the ten chromosomes of *P. agathidicida* 3770. Tracks above depict copy number variants, where blue rectangles indicate duplications and red rectangles indicate deletions. *P. agathidicida* isolate numbers are displayed on the right.

In previous work, the genetic diversity of *P. agathidicida* among a collection of isolates from various locations in New Zealand was shown to be very low (~0.1% sequence diversity), indicating a near-clonal population (Guo et al., 2020a). However, an assessment of copy number variation (CNV), based on re-mapping of Illumina reads from the different isolates (including Pa3770) onto the complete *P. agathidicida* 3770 genome assembly (Supplementary Table S3), suggested there is some structural diversity between the isolate genomes, with partial aneuploidy potentially providing increased or decreased gene copy numbers in those regions (Figure 5). While there appear to be some CNVs that are common to many of the isolates, others stand out as being unique; for example, the higher copy number of chromosome 10 of *P. agathidicida* 3770. This highlights the value of a complete genome assembly for discerning structural genome diversity.

#### Gaining a genome-wide view of gene expression

**Figure 6:**
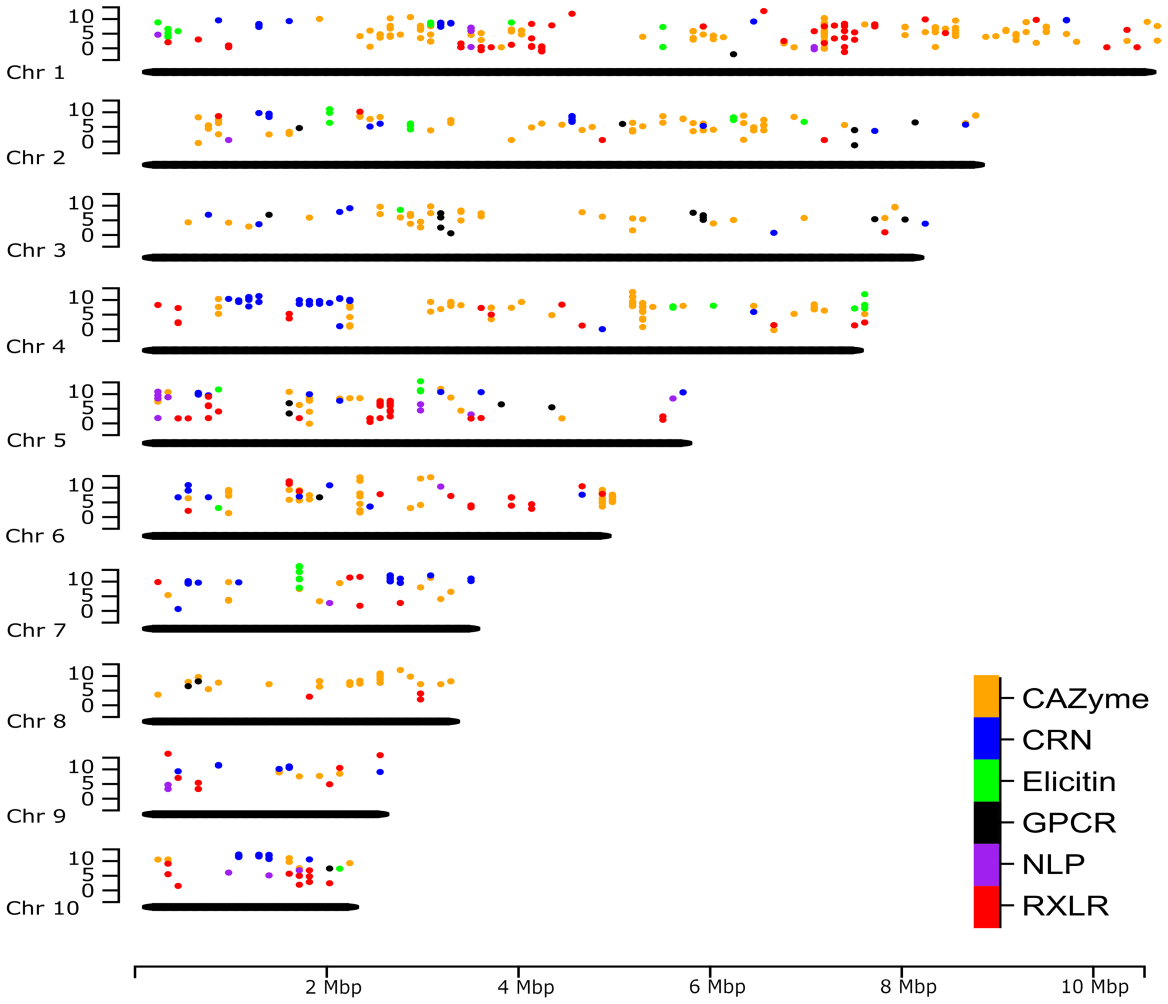
Genome-wide expression of putative effectors and other key gene classes. The positions of genes in six key gene classes in the *P. agathidicida* 3770 genome and the maximum expression value (log_2_ fragments per kilobase of exon per million mapped fragments [FPKM]) of those genes across a time course of expression on kauri roots at 6, 24, 48 and 72 h post-inoculation. Chr, chromosome.

A complete genome assembly also offers the opportunity to re-map previously obtained gene expression sequence reads to provide a genome-wide perspective. Unpublished RNA-seq data of *P. agathidicida* isolate 3813 (BioProject PRJNA864064), grown in kauri leaves and roots over a time course of infection, were mapped to the new *P. agathidicida* 3770 gene models. Isolate *P. agathidicida* 3813 is very similar in sequence to *P. agathidicida* 3770; between these two isolates, only 10,435 SNPs were identified across the whole genome, with just 2,140 of these falling in gene-coding regions, providing confidence in the accuracy of mapping *P. agathidicida* 3813 reads onto the *P. agathidicida* 3770 genome. Levels of gene expression were evaluated for genes in the six gene classes mentioned above. Figure 6 shows the positions of these genes across the genome and their maximum expression values (at any time point) *in planta*. From this genome-wide perspective, the positions of hotspots of gene expression can be determined for several of the gene groups. For example, there are clusters of highly expressed CAZyme and RXLR genes on chromosome 1, elicitin genes on chromosome 7 and a region of high CRN gene expression on chromosome 4. Supplementary Figure S5 shows a similar plot except showing positions of genes that are significantly up-regulated *in planta* compared to in culture.

#### Examining association of transposable elements with multi-copy CRN genes

In *P. infestans*, many of the large number of CRN genes are in repeat-rich regions and it was suggested that expansion of CRN gene families was facilitated by non-allelic homologous recombination involving repeats (Haas et al., 2009). A completely assembled genome can provide a clear picture of all or most of the repetitive elements and their locations with respect to protein-coding genes, facilitating studies of repeat-driven adaptation.

**Figure 7:**
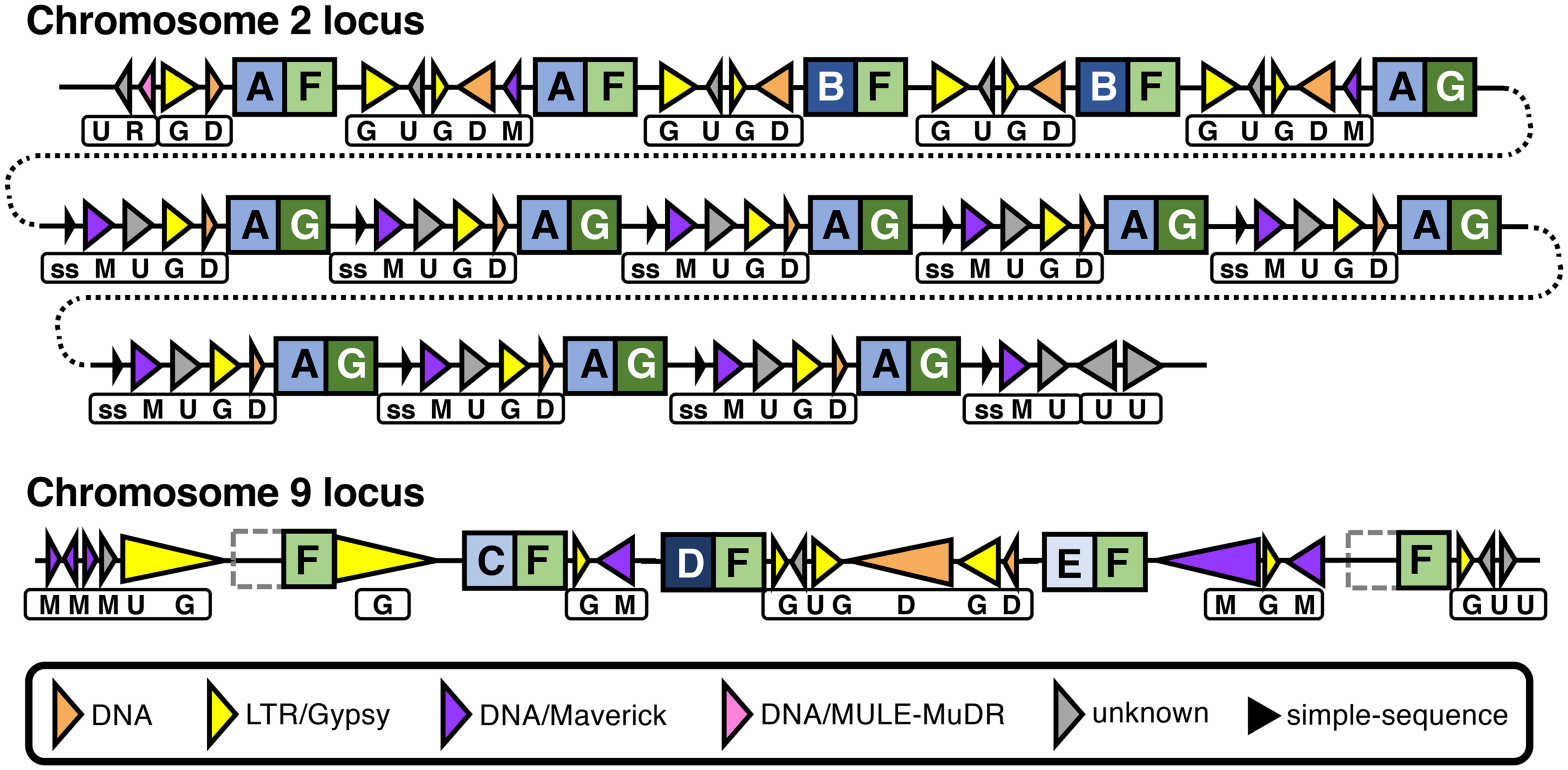
Recombination and repeats in two CRN gene clusters in the *Phytophthora agathidicida* genome. Genomic organization of CRN gene repeat clusters on chromosomes 2 and 9. CRN genes are indicated by square boxes with letters inside. N and C termini of predicted CRN proteins are indicated by blue and green boxes, respectively, with different shading and letters indicating different variants. Repeats are indicated by triangles, with transposons depicted as colored triangles and simple-sequence (ss) repeats as smaller, black triangles, as shown in the key. Triangle direction refers to the strand each repeat is encoded on. The different combinations of repeats between CRN genes are also indicated beneath by boxes with letters. The chromosome 2 cluster is shown over multiple lines (joined by dotted lines) for ease of visualization. In the chromosome 9 cluster, the left- and right-most CRN genes both encode proteins that lack the N-terminal domain, as indicated by grey dotted boxes. Diagram is not to scale, although large differences in repeat length are indicated schematically by small differences in triangle length.

Although *P. agathidicida* has a relatively large number of CRN genes, many of them are grouped in gene clusters of various sizes (Figure 3; Supplementary Table S2). The clustered genes occur most often in repeated head-to-tail orientation, suggestive of tandem gene duplication events, sometimes with one or more other types of genes separating them. In an interesting example, 13 adjacent CRN genes in a cluster on chromosome 2 are separated from each other by repeating sets of repeats including DNA/Maverick and LTR/Gypsy transposons (Figure 7). Maverick repeats are in the same class of DNA transposons as helitrons, which were reported as being closely associated with a similar CRN gene cluster in *P. infestans* (Haas et al., 2009). CRNs are modular proteins with a recombination hotspot between the N and C termini at a conserved HVLVXXP motif (Haas et al., 2009; Amaro et al., 2017). The 13 clustered *P. agathidicida* CRN genes fall into three sub-groups based on differences in their predicted N- and C-terminal domain sequences (Figure 7; Supplementary Figure S6). These shared repeats and protein domain swaps suggest the cluster has originated *via* recombination and duplication events, a potential sequence of which is shown in Supplementary Figure S7. The nine identical genes (*CRN31-39*) labelled ‘AG’ in Figure 7 had maximum *in planta* expression values of only 40–65 FPKM (fragments per kilobase of exon per million mapped fragments), whilst the ‘AF’ (*CRN27-28*) and ‘BF’ (*CRN29-30*) types all showed higher expression values (between 196–273 FPKM; Supplementary Table S4).

On chromosome 9, a set of three adjacent CRN genes (*CRN119-121*) are all different from one another in the predicted N-terminal region (Figure 7; Supplementary Table S4; Supplementary Figure S6) but were predicted to have identical C termini apart from one amino acid. This is again suggestive of recombination at the same CRN gene hotspot. Moreover, these genes are interspersed with similar repeat elements as found between the 13 CRN genes on chromosome 2, and they also share an identical C-terminal amino acid sequence (F) with those from some of the chromosome 2 CRNs. Indeed, a total of 31 CRN genes in the genome encode proteins that have a C-terminus with at least 99.8% amino acid identity to what we have denoted as type F, along with another 12 genes that were not called as CRNs but appear to be associated with CRN gene clusters (Supplementary Table S5). Two of these 12 additional genes were found flanking the three-CRN gene cluster on chromosome 9; the upstream gene (*CRN-like1*) encodes a protein that lacks the complete N-terminal region and 100 amino acids of the C-terminus, while the downstream gene (*CRN-like2*) encodes a protein that lacks almost all of the N-terminal region (Supplementary Table S4). All five CRN and CRN-like genes on chromosome 9 shown in Figure 7 had maximum *in planta* expression values between 224 and 292 FPKM (Supplementary Table S4). Together these results suggest both intra- and inter-chromosomal recombination and duplication events have occurred in the *P. agathidicida* genome and that in some cases gene expression is affected.

#### Seeking evidence for adaptation in multi-copy RXLR genes

Of the 147 candidate RXLR genes in the *P. agathidicida* 3770 genome, 30 occur in one of 11 families with identical or near-identical genes and are located on chromosomes 1, 5, 6 or 9 (Table 1; Supplementary Table S6). Most of these gene families consist of two or more identical genes and many have other genes, sometimes a different RXLR gene, between them (Table 1). The largest family, on chromosome 1, is a set of nine tandemly repeated genes (*RXLR29-1* to *29–9*) which are identical in sequence, suggesting recent duplication events. In the previous genome annotation, only one of these nine *RXLR29* genes was annotated (Guo et al., 2020a), highlighting the value of a fully-assembled genome for identifying families containing identical genes. Between each of the *RXLR29* genes is a repeated sequence of an unknown repeat class (Supplementary Table S1). RXLR29 was previously shown to trigger plant cell death when delivered to *Nicotiana tabacum* by *Agrobacterium tumefaciens*-mediated transient transformation, suggesting it is recognised by immune receptors in the plant (Guo et al., 2020a). Curiously, based on the gene expression studies outlined in Section “Gaining a genome-wide view of gene expression,” none of these nine genes are expressed *in planta* or in culture.

Besides RXLR29, genes in six of the 11 other RXLR gene families also appear to be silenced, including gene families *RXLR4*, *11*, *36*, *46, 120* and *144*, located on chromosomes 1, 5, 6 and 9 (Table 3; Supplementary Table S6). In contrast, the *RXLR106* family, which consists of two identical genes, appears to be among the most highly expressed of the RXLR genes; this is the only identical RXLR gene pair that is located on two separate chromosomes (1 and 9), although, based on RNA-seq read mapping, it is not possible to tell whether only one or both of these identical genes are actually expressed. However, control of individual gene expression is also important, as seen on chromosome 9 where one of the most highly expressed singleton RXLR gene candidates (*RXLR53*) is located less than 3 kb from a CRN gene (*CRN128*) which is expressed at very low levels (Supplementary Table S2). A complete genome assembly offers great potential to study gene regulation on a genome level and the roles of certain classes of genome repeat elements and epigenetic factors provide a rich area for discovery.

**Table 3:** Candidate RXLR gene families in the *Phytophthora agathidicida* genome.

Chromosome	Position^a^	RXLR family (copy number)^b^	Gene orientations	Expression *in vitro*^c^	Highest expression *in planta*^c^	Other genes between RXLR genes
1	818,045	*RXLR144* (2)	− +	0	0	0
1	3,205,298	*RXLR29* (9)	all +	0	0	0
1	3,909,826	*RXLR11* (2)	− +	0	0	34
1	6,969,331	*RXLR77* (2)	+ −	+	+++	1
1	7,188,628	*RXLR27* (2)	− +	+	+++	9
1	7,498,520	*RXLR132* (2)	− −	++	+++	7
5	232,263	*RXLR36* (3)	+ − +	0	0	2 and 16
5	2,372,879	*RXLR56* (2)^d^	− +	0	++	12
5	5,316,559	*RXLR4* (2)	− +	+	0	0
6	3,400,991	*RXLR120* (2)	+ −	0	+	0
9	450,340	*RXLR46* (2)	+ −	+	0	12
1 & 9	6,326,548, 138,119	*RXLR106* (2)	+ −	+	++++	Different chromosomes

a Position denotes the first nucleotide in the coding sequence for the first gene in the family.

b See Supplementary Table S6 for details.

c Bands of maximum mean gene expression (fragments per kilobase of exon per million mapped fragments [FPKM]) in vitro or in planta indicated as 0 (<1), + (>1), ++ (>10), +++ (>100), ++++ (>1,000).

d non-synonymous single nucleotide polymorphism (SNP) D18G in one gene copy.

## Discussion

In this study, we combined data from long-read PacBio sequencing and Hi-C chromatin conformation capture to assemble the genome of the kauri dieback pathogen *Phytophthora agathidicida* to chromosome level. The assembly of the genome into ten chromosomes is a considerable improvement over the previous assembly of 3,689 scaffolds, which was derived using short-read Illumina sequences (Studholme et al., 2016). The newly generated genome has an estimated size of 57 Mb and appears to be accurate based on the high (100%) BUSCO score obtained using the genome assembly and the Stramenopile lineage gene set. The genome size is thus 20 Mb larger than that predicted in the previous assembly based on short read sequencing (Studholme et al., 2016). Because short-read assemblies often miss or collapse long repetitive regions, it seems likely that much of this extra 20 Mb comprises repeat sequences such as transposable elements. However, some members of gene families with identical or near-identical adjacent genes were also missed in the earlier assembly, such as the *RXLR29* gene family.

Long-read technologies have been used for sequencing other *Phytophthora* genomes, including *P. cactorum* (Yang et al., 2018), *P. capsici* (Stajich et al., 2021), *P. cinnamomi* (Engelbrecht et al., 2021) and *P. ramorum* (Carleson et al., 2022), although none of these genomes were assembled to chromosome level and none of these authors reported using Hi-C data. In our work, whilst the Hi-C data enabled us to join a few sub-chromosomal scaffolds together, it was the availability of extremely high coverage long reads overlapping the junctions that primarily enabled us to complete the assembly. The Hi-C data did, however, confirm the overall assembly as well as indicate the centromere positions. The validity of the centromere positions was supported by the presence of LTR/Copia (CoLT) retrotransposon elements which have been associated with centromeres in other *Phytophthora* species (Fang et al., 2020).

Factors that likely influenced our ability to assemble the *P. agathidicida* genome to chromosome level include its relatively small genome size (57 Mb), low repeat content (34%), and very low heterozygosity (0.02%). The 57 Mb genome size of *P. agathidicida* is similar to that of *P. ramorum* (Carleson et al., 2022) but much smaller than those of *P. capsici* (94 Mb; Stajich et al., 2021), *P. cinnamomi* (107 Mb; Engelbrecht et al., 2021), *P. sojae* (95 Mb; Tyler et al., 2006) and *P. infestans* (240 Mb; Haas et al., 2009). In general, these differences in genome size reflect the repeat content (Engelbrecht et al., 2021) and the higher the repeat content the more challenging it is to assemble a complete genome. The heterozygosity level of 0.02% is considerably lower than those estimated for other *Phytophthora* species for which the same method was used, for example *P. cinnamomi* (1.36%; Engelbrecht et al., 2021), *P. infestans* (0.695%; Fletcher and Michelmore, 2018), *P. chlamydospora* (0.68%), *P. gonapodyides* (1.88%) and *P. pseudosyringae* (0.15%; McGowan et al., 2020).

Although chromosome-level assemblies of *Phytophthora* species are currently rare, repeat-rich genomes of fungal pathogens have been fully assembled using long-read and Hi-C sequence data. For example, the 67 Mb genome sequence of the fungal tomato pathogen *Cladosporium fulvum* (now called *Fulvia fulva*) was assembled to 14 chromosomes using these technologies (Zaccaron et al., 2022), representing a considerable advance over the 4,865 scaffolds of the earlier assembly, albeit from a different isolate of this species (de Wit et al., 2012). However, the 1 Gb genome sequence of the fungal myrtle rust pathogen *Austropuccinia psidii*, of which 91% is repetitive sequence, was assembled into 66 scaffolds rather than to chromosome-level (Tobias et al., 2021), highlighting the ongoing limitations to complete assembly imposed by repeats.

The *P. agathidicida* genome assembly provides a unique insight into how a *Phytophthora* genome is constructed. The high level of synteny with the chromosome-level assembly of the oomycete *Peronospora effusa* (Fletcher et al., 2021) and the 70-contig assembly of *P. sojae* (Fang et al., 2020) suggests conservation of high-level genome organisation, despite low sequence identity, and may assist in assembly of other oomycete genome sequences. Comparisons among the ten chromosomes of *P. agathidicida* revealed only minor variations in gene density, GC content and percentage of repeats, although some classes of genes, among the six classes we studied, were more prominent on some chromosomes than others. Based on our analyses, this asymmetric distribution of genes across the chromosomes was most likely due to physical clustering of genes in gene families. We found multicopy gene families encoding RXLRs and CRNs in *P. agathidicida* that most likely occurred due to gene duplication, in keeping with effector gene expansion observed in other *Phytophthora* species such as *P. infestans* (Haas et al., 2009), *P. sojae* (Tyler et al., 2006) and *P. cactorum* (Yang et al., 2018). This phenomenon can explain the irregular distribution of some of the gene classes on *P. agathidicida* chromosomes. For example, chromosome 10 has a relatively large number of CRN genes (17) for its small size but most of these occurred in two clusters of five and 11 genes. Chromosome 10 does, however, have some special features compared to the other chromosomes: lower gene density, higher repeat content (51%), increased copy number suggestive of partial aneuploidy and a higher proportion of SNPs among a set of New Zealand *P. agathidicida* isolates, particularly in protein-coding regions (Table 2). However, as chromosome 10 was present in all the other *P. agathidicida* isolates tested and because it contains the rDNA locus, it does not appear to be a dispensable or accessory chromosome. Such dispensable or accessory chromosomes are often found in fungi. This includes *Fulvia fulva*, where only five of 24 isolates examined were found to contain the smallest chromosome, chromosome 14 (Zaccaron et al., 2022).

The chromosome-level assembly of the *P. agathidicida* genome also provides high confidence for identifying gene locations relative to other features, including where there are multicopy genes in gene families and repetitive elements such as transposons. It is known that *Phytophthora* effector genes, such as RXLRs, are often found in gene-poor, repeat-rich regions of the genome, where the proximity of the repeats provides opportunities for rapid adaptation and evolution by processes such as gene duplication and recombination (Haas et al., 2009). An often-used method to assess the gene density surrounding certain gene classes is to assess the intergenic distance to the next gene. As found in *P. cinnamomi* (Engelbrecht et al., 2021), candidate CRN, RXLR and NLP genes of *P. agathidicida* were significantly enriched within gene-poor regions (Figure 4). The CAZyme genes showed a significant but relatively small median distance from other genes, perhaps influenced by the broad diversity of CAZyme classes we considered. Although we found evidence to support a compartmentalized genome in *P. agathidicida*, further work is required to determine if rates of evolutionary change differ between the two types of compartments, as was found in *P. infestans* (Raffaele et al., 2010).

Given the expansion and diversification of effector genes that occur in many *Phytophthora* pathogens (Haas et al., 2009; McGowan and Fitzpatrick, 2017), a deeper understanding of how these pathogens adapt to invade and subdue their hosts will require analysis of evolutionary processes occurring in multi-gene families. Where these gene families are comprised of tandem repeats of near-identical genes, high-quality genome assemblies based on long-read sequencing technology provide a clearer picture compared to fragmented assemblies from which some genes may be missing. As examples, we looked at regions in the *P. agathidicida* genome containing tandemly repeated CRN and RXLR gene family candidates.

*P. agathidicida* appears to have a relatively large number of CRN genes (145) which will form the basis for future studies. CRN proteins are of great interest in that they are highly conserved, being found in all plant-pathogenic oomycetes studied so far (Amaro et al., 2017). CRNs tend to have a conserved N-terminal domain with two conserved motifs (LXLFLAK and HVLVVVP) but a variable C-terminal functional domain which denotes their classification (Zhang et al., 2016). Most CRNs are expressed both in culture and *in planta* (Haas et al., 2009; Amaro et al., 2017). In the case of *P. capsici* in tomato, there were two patterns of gene expression with (a) upregulation in both early and late stages of infection and (b) a gradual increase from early to late stages (Stam et al., 2013). Despite their name, not all CRNs induce cell death in plants and some can suppress cell death (Liu et al., 2011; Amaro et al., 2017).

In the *P. agathidicida* genome, transposable DNA elements (such as DNA/Maverick), were associated with clusters of CRN genes, along with LTR/Gypsy and other repetitive elements. Some types of CRN genes were likewise associated with DNA elements (helitrons) in *P. infestans* (Haas et al., 2009). Helitrons duplicate by rolling circle replication and can mediate genome evolution by promoting duplication and recombination (Kapitonov and Jurka, 2007), leading to the suggestion that CRN evolution in *P. infestans* is mediated by helitrons (Haas et al., 2009). A gypsy retrotransposon was also found in the C-terminal domain of a CRN-coding gene, *PITG_23144*, in *P. infestans* (Haas et al., 2009). Inspection of two clusters of CRN genes in the *P. agathidicida* genome provided evidence for recombination, including domain swaps, at the ‘hotspot’ junction of the N- and C-terminal domains. One of the CRN gene clusters contained several tandem identical repeats, indicative of recent duplication events, whilst the other cluster was flanked by CRN-like genes that lacked most or all of the N-terminal domain. In addition to recombination/duplication, our results also suggest recent transposon activity in *P. agathidicida* based on low divergence of transposon sequences (Supplementary Figure S4), as also shown in *P. infestans* (Haas et al., 2009). A more than three-fold difference in gene expression was seen between different types of recombinant CRN paralogs in the larger *P. agathidicida* cluster we investigated, suggesting a possible functional consequence of transposon-mediated recombination. The evolution of CRN genes and the roles of transposable and other repetitive elements in this process is a fascinating area for further study. Expansion of CRN genes appears to be associated with species that include necrotrophy in their life cycle (Stam et al., 2013). Due to the hemibiotrophic nature of most *Phytophthora* species (Boevink et al., 2020), the CRN genes may be a focal point for adaptation in these pathogens, including *P. agathidicida*.

Almost twice as many RXLR genes (147) were identified in the new *P. agathidicida* genome assembly compared to the previous fragmented assembly (78). RXLRs are the best studied among all the effectors of *Phytophthora* species (Anderson et al., 2015; Deb et al., 2018; Wang et al., 2019a; Chepsergon et al., 2021) and are often the largest class of intracellular effectors in *Phytophthora* pathogens (Boevink et al., 2020; Midgley et al., 2022). RXLR proteins function in many different regions of the cell and have many different roles (Boevink et al., 2020; Midgley et al., 2022). Among these, the ability of some RXLRs to induce or suppress plant cell death are well known (Deb et al., 2018). For example, in *P. infestans* the RXLR effector Avr3A elicits cell death in potato when recognised as an avirulence factor (Armstrong et al., 2005). In our earlier work on *P. agathidicida*, we identified eight RXLR candidates that elicited cell death in the non-host *Nicotiana* spp. and one (PaRXLR40) that suppressed RXLR-induced cell death (Guo et al., 2020a). One of those cell-death eliciting RXLR candidates, RXLR29, was encoded by a gene that formed part of a cluster of nine identical genes when we examined the new chromosome-level genome assembly. Thus the increased RXLR numbers in the new *P. agathidicida* genome assembly are partly accounted for by multicopy gene families that were undetected in the earlier fragmented genome.

The nine gene copies in the *P. agathidicida* cell-death eliciting RXLR29 cluster are separated by repeats of an unknown class that could potentially have facilitated gene duplication. However, whilst gene duplication and divergence are known as drivers of adaptation (Goss et al., 2013), none of the nine *RXLR29* copies were expressed during infection of kauri. Similarly six of the 11 other RXLR gene clusters in the *P. agathidicida* genome were silenced or expressed at very low levels (Supplementary Table S6). Silencing of effector gene expression is a well-established adaptation mechanism whereby pathogens can avoid recognition by a plant host, which could otherwise provoke a defence response, often including localised cell death (Qutob et al., 2013; Wang et al., 2019b, 2022). In contrast, the nine identical and four near-identical CRN genes on chromosome 2 (Figure 7) were clearly expressed (Supplementary Table S4), supporting the reliability of our RNA-seq mapping method to determine expression in identical multi-copy genes.

In agricultural systems, the identification of effectors that elicit host defence responses has enabled the screening and breeding of resistant cultivars (Vleeshouwers and Oliver, 2014; Chepsergon et al., 2021). Effectors of gymnosperm pathogens, both fungal and oomycete, have been shown to elicit defence responses in model angiosperm hosts, suggesting similar mechanisms in angiosperm and gymnosperm plant-pathogen interactions (Chen et al., 2015; Guo et al., 2020a,b; Hunziker et al., 2021; Tarallo et al., 2022). Gymnosperm trees such as conifers have diverse sets of immune receptors, some of which respond to drought as well as to pathogens (Van Ghelder et al., 2019). A recent study showed that the defence responses of disease-tolerant *Pinus contorta* included increased expression of genes in PAMP- and effector-triggered immunity pathways as well as of certain immune receptors in response to infection with the fungal pathogen *Dothistroma septosporum* (Lu et al., 2021). Thus, the study of effectors in oomycete forest pathogens has the potential to identify resistance traits in gymnosperm trees such as kauri. However, pathogens can quickly adapt by silencing, mutating or deleting their genes. In *Phytophthora* species, insights into adaptation of virulence traits have been gained by studying chromatin-level modification of gene expression (Wang et al., 2020b), diversifying selection (Zhang et al., 2019) and loss of heterozygosity (Lamour et al., 2012; Dale et al., 2019). A focus on core effectors that elicit defence responses offers the promise of more durable resistance; these are effectors that are highly conserved, highly expressed *in planta* and essential for virulence or pathogen growth such that loss of function would be detrimental to the pathogen (Chepsergon et al., 2021).

In some cases RXLRs that elicit cell death at a later stage of infection, such as *P. capsici* PcAvh1 (Chen et al., 2019), might facilitate the transition from biotrophy to necrotrophy. In contrast, *P. sojae* PsAvh52 suppresses cell death in early stages of infection in soybean and is required for full virulence (Li et al., 2018). Thus some RXLRs play a key role in orchestrating the pathogen lifestyle during both biotrophic and necrotrophic stages. The study of RXLRs with different roles such as these in *P. agathidicida* is likely to reveal important virulence factors that could be targets for disease control.

In the case of trees such as kauri that can live for over a thousand years, it would be naive to think that a single mechanism will achieve durable resistance. Disease resistance or tolerance needs to encompass a diversity of mechanisms and function within a broader ecological setting that includes the effects of soil, companion plants and, importantly, potential protective effects of the microorganisms that naturally live in, on and around the tree (i.e., the host holobiont) (Pautasso et al., 2015; Padamsee et al., 2016; Lewis et al., 2019; Terhonen et al., 2019; Wang et al., 2019b; Byers et al., 2020; Wang et al., 2020a). However, by increasing our knowledge of the molecular interactions between *Phytophthora* pathogens and their hosts, in a broad ecological setting, we stand a better chance of protecting those plant hosts against the devastating effects of disease. The complete genome sequence of *P. agathidicida* provides a valuable stepping stone towards a better understanding of these plant destroyers.

## Data availability statement

The datasets presented in this study can be found in online repositories. The names of the repository/repositories and accession number(s) can be found at: https://www.ncbi.nlm.nih.gov/, PRJNA734652; https://www.ncbi.nlm.nih.gov/, PRJNA864064.

## Author contributions

RB, MC, RM, NG, NW, and CM conceived and guided the study. YG, DW, DS, NC, EB, AG, MG, RL, PP, JS, RB, and MC designed and performed experiments and analyzed data. RB and MC led manuscript writing. All authors contributed to the article and approved the submitted version.

## Funding

This research was funded by the New Zealand Ministry of Business, Innovation and Employment (Ngā Rākau Taketake – Myrtle Rust and Kauri Dieback Research, grant number C09X1817, and Healthy Trees Healthy Future Programme, grant number C04X1305) and the New Zealand Tertiary Education Commission (Bioprotection Aotearoa, grant number 39504).

## Conflict of interest

The authors declare that the research was conducted in the absence of any commercial or financial relationships that could be construed as a potential conflict of interest.

## Publisher’s note

All claims expressed in this article are solely those of the authors and do not necessarily represent those of their affiliated organizations, or those of the publisher, the editors and the reviewers. Any product that may be evaluated in this article, or claim that may be made by its manufacturer, is not guaranteed or endorsed by the publisher.

## Footnotes

^1^ https://github.com/PacificBiosciences/IsoSeq

^2^ https://grunwaldlab.github.io/annot_toolbox/complement_gff/

^3^ https://mpcox.github.io/2D_FIR_distribution

^4^ https://mpcox.github.io/3D_contact_distribution

^5^ http://broadinstitute.github.io/picard/

^6^ https://github.com/abyzovlab/CNVpytor
