# Supplementary material for: Chromosome-level assembly of the Phytophthora agathidicida genome reveals adaptation in effector gene families
Source: Front Microbiol. 2022 Nov 2;13:1038444. doi: 10.3389/fmicb.2022.1038444 (PMC9667082; doi:10.3389/fmicb.2022.1038444)
Supplement: Supplementary file 1 [file Data_Sheet_1.PDF]

Supplementary Material - Figures 1–7 and Supplementary Data 1.

Supplementary Figure S1 BUSCO analysis of genome assemblies based on long-read sequences.

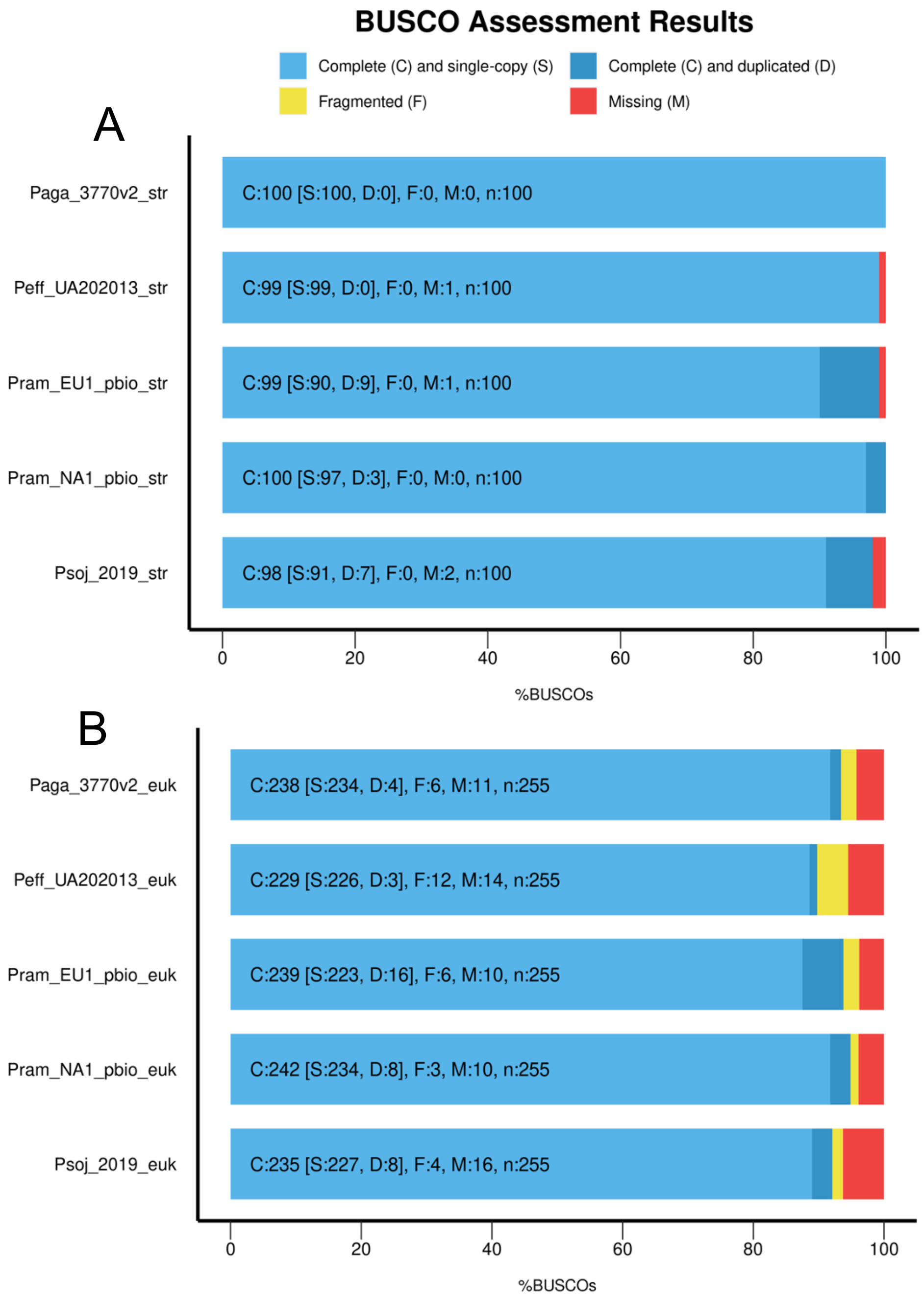

BUSCO v4.14 (Benchmarking Universal Single-Copy Orthologs) was used in genome mode with (A) stramenopile lineage and (B) Eukaryota gene sets from OrthoDB, using default parameter values. Oomycete genome assemblies based on long-read sequence data are *Phytophthora agathidicida* (Paga; this study), *Peronospora effusa* (Peff; Fletcher et al., 2021), *Phytophthora ramorum* (Pram; Carleson et al., 2022) and *Phytophthora sojae* (Psoj; Fang et al., 2020).

Supplementary Figure S2 Genome profiling.

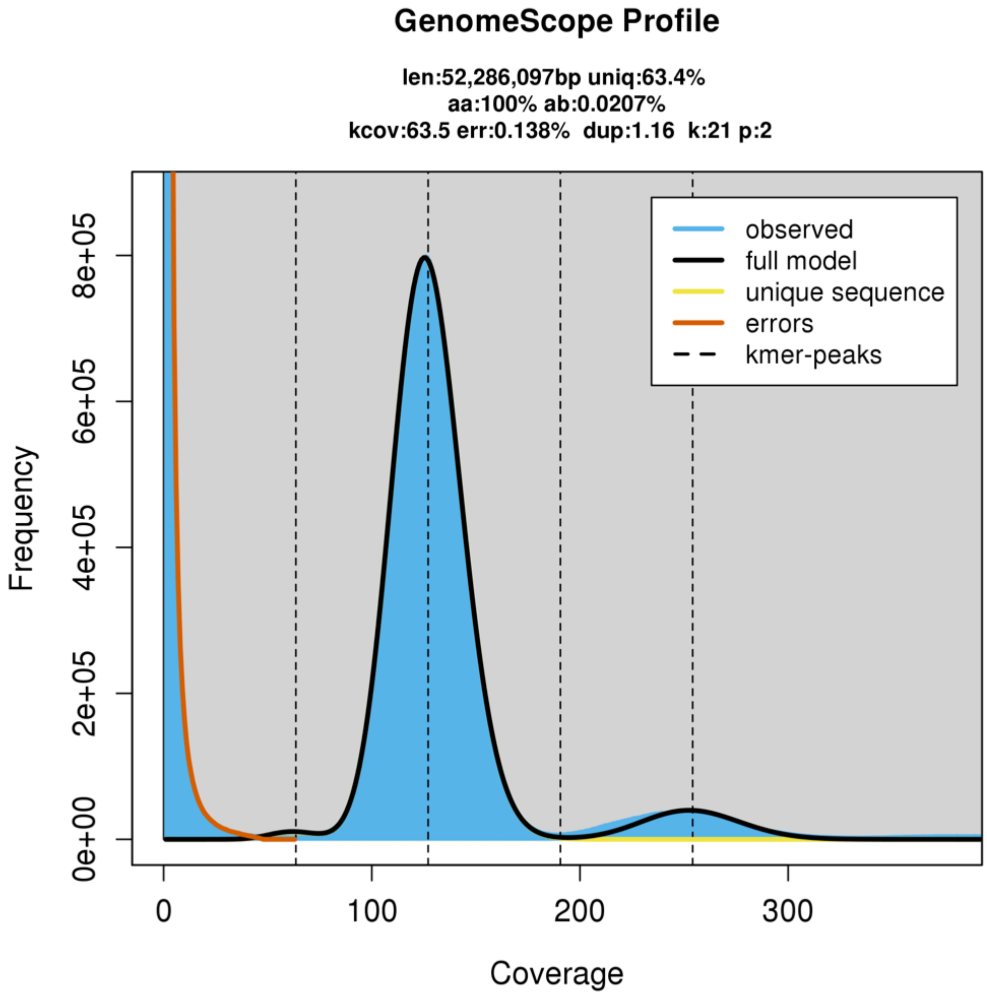

Genomic profiling using Genomescope and short-read data for the *Phytophthora agathidicida* 3770 genome. This analysis suggested this isolate is diploid with an estimated genome size of 52.3 Mb (slightly smaller than the actual assembly size of 57 Mb) and to have genome-wide heterozygosity of 0.0207%. The small 1n peak at 63.5 is very small, concordant with the very low heterozygosity.

Supplementary Figure S3 Repeat classes in the genome.

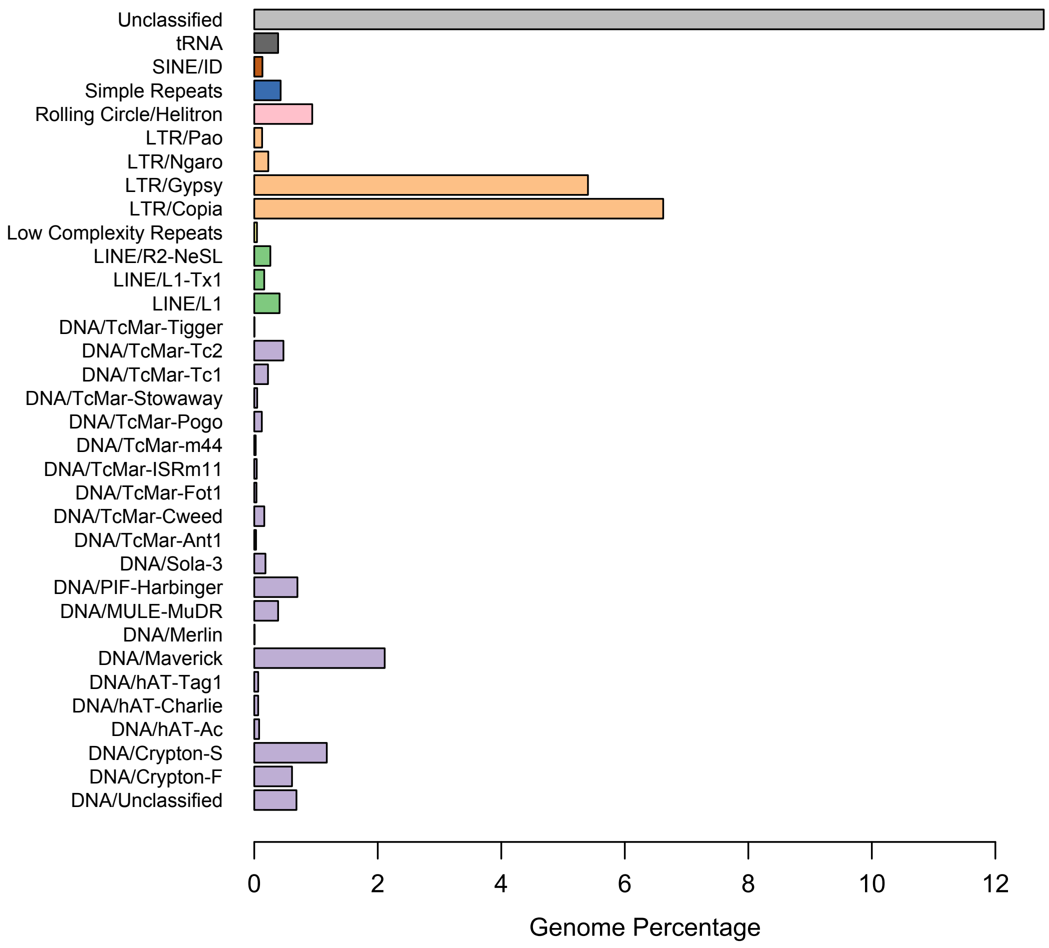

The percentage of the *Phytophthora agathidicida* 3770 genome taken up by different repeat classes.

Supplementary Figure S4. Transposable element activity.

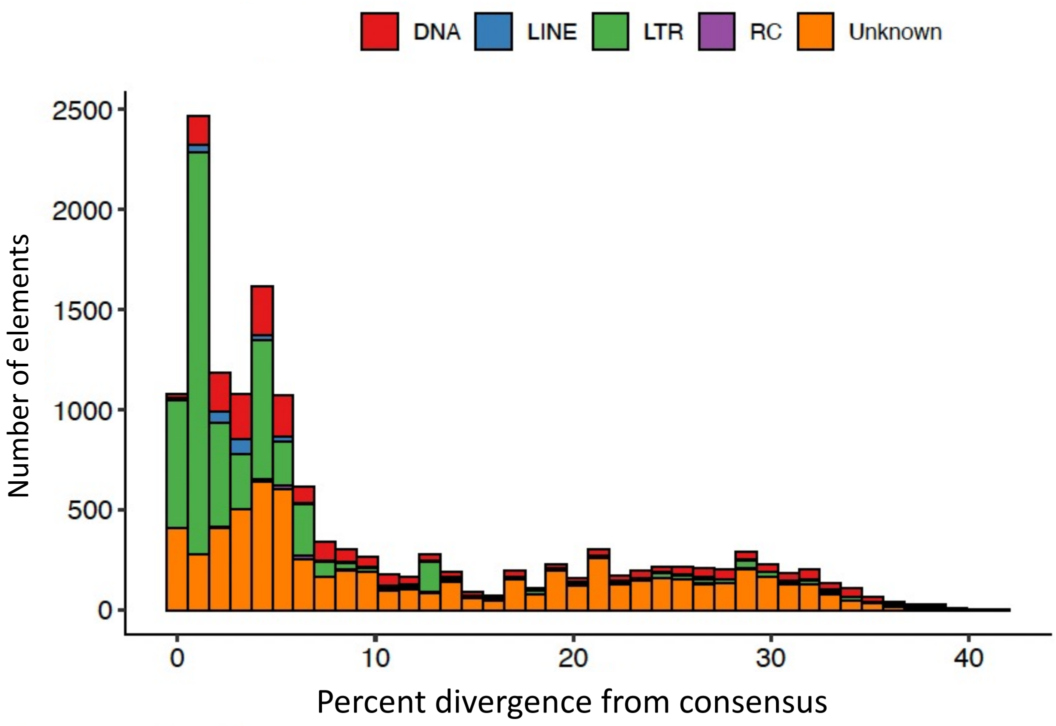

Transposable element activity in the *Phytophthora agathidicida* 3770 genome, based on branch lengths in a phylogeny of transposable element (TE) sequences. Divergence of LTR copies from their consensus sequence estimates the timeline of TE insertions. TE classes are represented by shading following the figure legend where DNA = DNA elements, LINE = long interspersed nuclear elements, LTR = long terminal repeat elements, RC = rolling circle transposons and Unknown = multi-copy sequences that could not be assigned to a TE class. The strong skew towards low divergence from the consensus suggests recent activity, especially in LTRs.

Supplementary Figure S5. Genome wide up-regulation of putative effector genes.

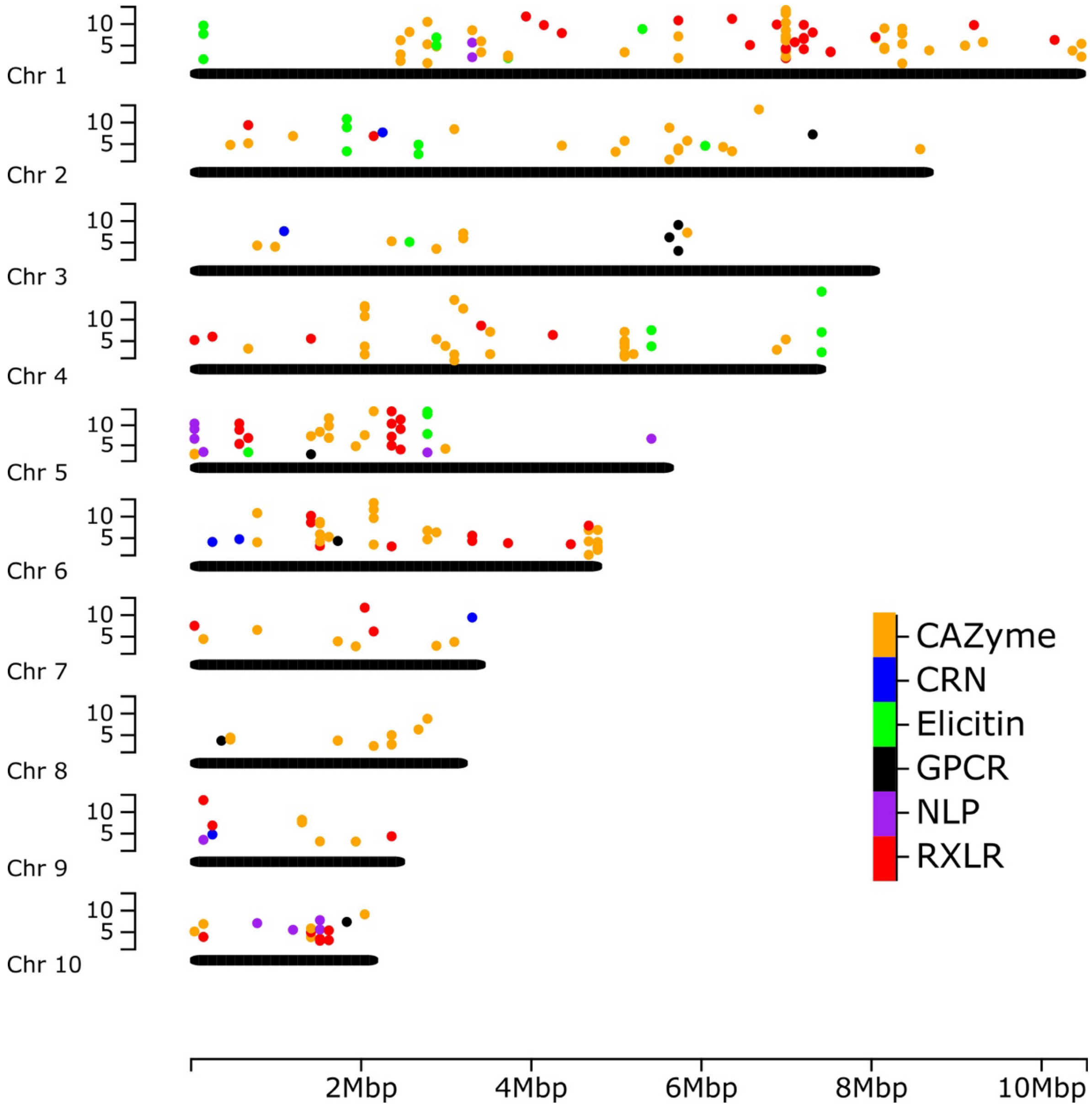

Genomic positions of effectors and other gene classes in the *P. agathidicida* 3770 genome that are upregulated during infection of kauri roots compared with *in vitro* growth. The positions of upregulated genes in six key gene classes are indicated and the maximum fold change ($\log_2$ FPKM) value from a time course of infection on kauri roots at 6, 24, 48 and 72 hours post-inoculation of each of these genes is shown. Chr, chromosome.

Supplementary Figure S6. Alignment of amino acid sequences for selected CRN proteins.

A) Alignment of amino acid sequences for 13 CRN proteins (CRN27–39) encoded by clustered genes on chromosome 2 (commencing at nucleotide 4,379,131). Shading indicates conservation.

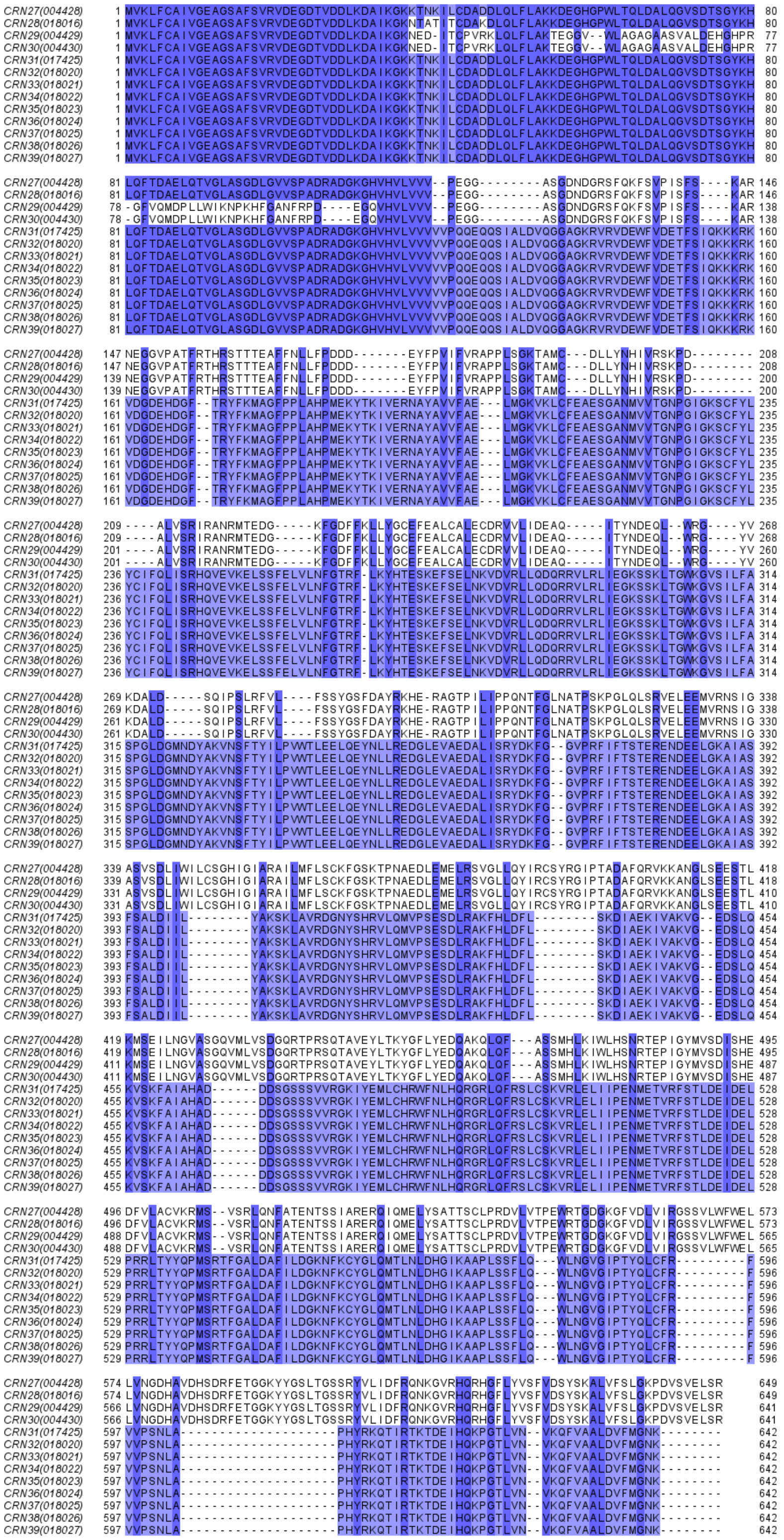

B) Alignment of amino acid sequences for three CRN proteins (CRN119–121) and two CRN-like proteins (truncated CRN proteins missing all or most of their N-terminal region) encoded by clustered genes on chromosome 9 (commencing at nucleotide 6,421,40). Shading indicates conservation.

```
CRN-like-1(017816)      ---------------------------------------------------------------------------------
CRN119(016136)        1 MVLLNCAIIGE-GSVISIIIEEWKTVALLKEAIKEAKKNDLKDVDADKLQLFLAKTEGGAWLVSNSPDVISMRSGAIPEHV  80
CRN120(016137)        1 MVKLFCAIVGEAGSAFSVEVDEAASVDDLKKAIKGKNED-IT-CPVRKLQLFLAKTGNGAWLTENEVAEGVIDTSGLKPLN  79
CRN121(017817)        1 MMTLYCAIVGEAGSAFPVDINAGLSVGHLKDAIKGKKTNKIL-CDADDLQLFLAKTEGGAWLQSRSEDVKKLKKGEKTALI  80
CRN-like-2(017818)      ---------------------------------------------------------------------------------

CRN-like-1(017816)      ---------------------------------------------------------------------------------
CRN119(016136)       81 KKL--LNEEIDPTEEIGDLFGGA-----PTKKVIHVLVVVPEGGASGDNDGRSFQKFSVPISFSKARNEGGVPATFRTHRS 154
CRN120(016137)       80 VARAKLRRVGLSNEQVGEVDEVDEEEEAAGNGPVNVLVVVPEGGASGDNDERSFQKFSVPISFSKARNEGGVPATFRTHRS 160
CRN121(017817)       81 EALTHEDRELQGEDGLAEVLTGMDQ---PFTGQIHVLVVVPEGGASGDNDERSFQKFSVPISFSKARNEGGVPATFRTHRS 158
CRN-like-2(017818)    1 ----------------------MDQ---PSTGQIHVLVVVPEGGASGDNDERSFQKFSVPISFSKARNEGGVPATFRTHRS  56

CRN-like-1(017816)    1 ----------------------------------------------------------MTEDGKFGDFFKLLYGCEFEALC  23
CRN119(016136)      155 TTTEAFFNLLFPDDDEYFPVIFVRAPPLSGKTAMCDLLYNHIVRSKPDALVSRIRANRMTEDGKFGDFFKLLYGCEFEALC 235
CRN120(016137)      161 TTTEAFFNLLFPDDDEYFPVIFVRAPPLSGKTAMCDLLYNHIVRSKPDALVSRIRANRMTEDGKFGDFFKLLYGCEFEALC 241
CRN121(017817)      159 TTTEAFFNLLFPDDDEYFPVIFVRAPPLSGKTAMCDLLYNHIVRSKPDALVSRIRANRMTEDGKFGDFFKLLYGCEFEALC 239
CRN-like-2(017818)   57 TTTEAFFNLLFPDDDEYFPVIFVRAPPLSGKTAMCDLLYNHIVRSKPDALVSRIRANRMTEDGKFGDFFKLLYGCEFEALC 137

CRN-like-1(017816)   24 ALECDRVVLIDEAQITYNDEQLWRGYVKDALDSQIPSLRFVLFSSYGSFDAYRKHERAGTPILIPPQNTFGLNATPSKPGL 104
CRN119(016136)      236 ALECDRVVLIDEAQITYNDEQLWRGYVKDALDSQIPSLRFVLFSSYGSFDAYRKHERAGTPILIPPQNTFGLNATPSKPGL 316
CRN120(016137)      242 ALECDRVVLIDEAQITYNDEQLWRGYVKDALDSQIPSLRFVLFSSYGSFDAYRKHERAGTPILIPPQNTFGLNATPSKPGL 322
CRN121(017817)      240 ALECDRVVLIDEAQITYNDEQLWRGYVKDALDSQIPSLRFVLFSSYGSFDAYRKHERAGTPILIPPQNTFGLNATPSKPGL 320
CRN-like-2(017818)  138 ALECDRVVLIDEAQITYNDEQLWRGYVKDALDSQIPSLRFVLFSSYGSFDAYRKHERAGTPILIPPQNTFGLNATPSKPGL 218

CRN-like-1(017816)  105 QLSRVELEEMVRNSIGASVSDLIWILCSGHIGIARAILMFLSCKFGSKTPNAEDLEMELRSVGLLQYIRCSYRGIPTADAF 185
CRN119(016136)      317 QLSRVELEEMVRNSIGASVSDLIWILCSGHIGIARAILMFLSCKFGSKTPNAEDLEMELRSVGLLQYIRCSYRGIPTADAF 397
CRN120(016137)      323 QLSRVELEEMVRNSIGASVSDLIWILCSGHIGIARAILMFLSCKFGSKTPNAEDLEMELRSVGLLQYIRCSYRGIPTADAF 403
CRN121(017817)      321 QLSRVELEEMVRNSIGASVSDLIWILCSGHIGIARAILMFLSCKFGSKTPNAEDLEMELRSVGLLQYIRCSYRGIPTADAF 401
CRN-like-2(017818)  219 QLSRVELEEMVRNSIGASVSDLIWILCSGHIGIARAILMFLSCKFGSKTPNAEDLEMELRSVGLLQYIRCSYRGIPTADAF 299

CRN-like-1(017816)  186 QRVKKANGLSEESTLKMSEILNGVASGQVMLVSDGQRTPRSQTAVEYLTKYGFLYEDQAKQLQFASSMHLKIWLHSNRTEP 266
CRN119(016136)      398 QRVKKANGLSEESTLKMSEILNGVASGQVMLVSDGQRTPRSQTAVEYLTKYGFLYEDQAKQLQFASSMHLKIWLHSNRTEP 478
CRN120(016137)      404 QRVKKANGLSEESTLKMSEILNGVASGQVMLVSDGQRTPRSQTAVEYLTKYGFLYEDQAKQLQFASSMHLKIWLHSNRTEP 484
CRN121(017817)      402 QRVKKANGLSEESTLKMSEILNGVASGQVMLVSDGQRTPRSQTAVEYLTKYGFLYEDQAKQLQFASSMHLKIWLHSNRTEP 482
CRN-like-2(017818)  300 QRVKKANGLSEESTLKMSEILNGVASGQVMLVSDGQRTPRSQTAVEYLTKYGFLYEDQAKQLQFASSMHLKIWLHSNRTEP 380

CRN-like-1(017816)  267 IGYMVSDISHEDFVLACVKRMSVSRLQNFATENTSSIARERQIQMELYSATTSCLPRDVLVTPEWRTGDGKGFVDLVIRGS 347
CRN119(016136)      479 IGYMVSDISHEDFVLACVKRMSVSRLQNFATENTSSIARERQIQMELYSATTSCLPRDVLVTPEWRTGDGKGFVDLVIRGS 559
CRN120(016137)      485 IGYMVSDISHEDFVLACVKRMSVSRLQNFATENTSSIARERQIQMELYSATTSCLPRDVLVTPEWRTGDGKGFVDLVIRGS 565
CRN121(017817)      483 IGYMVSDISHEDFVLACVKRMSVSRLQNFATENTSSIARERQIQMELYSATTSCLPRDVLVTPEWRTGDGKGFVDLVIRGS 563
CRN-like-2(017818)  381 IGYMVSDISHEDFVLACVKRMSVSRLQNFATENTSSIARERQIQMELYSATTSCLPRDVLVTPEWRTGDGKGFVDLVIRGS 461

CRN-like-1(017816)  348 SVLWFWELLVNGDHAVDHSDRFETGGKYYGSLTGSSRYVLIDFRQNKGVRHQRHGFLYVSFVDSYSKALVFSLGKPDVSVE 428
CRN119(016136)      560 SVLWFWELLVNGDHAVDHSDRFETGGKYYGSLTGSSRYVLIDFRQNKGVRHQRHGFLYVSFVDSYSKALVFSLGKPDVSVE 640
CRN120(016137)      566 SVLWFWELLVNGDHAVDHSDRFETGGKYYGSLTGSSRYVLIDFRQNKGVRHQRHGFLYVSFVDSYSKALVFSLGKPDVSVE 646
CRN121(017817)      564 SVLWFWELLVNGDHAVDHSDRFETGGKYYGSLTGSSRYVLIDFRQNKGVRHQRHGFLYVSFVDSYSKALVFSLGKPDVSVE 644
CRN-like-2(017818)  462 SVLWFWELLVNGDHAVDHSDRFETGGKYYGSLTGSSRYVLIDFRQNKGVRHQRHGFLYVSFVDSYSKALVFSLGKPDVSVE 542

CRN-like-1(017816)  429 LSR 431
CRN119(016136)      641 LSR 643
CRN120(016137)      647 LSR 649
CRN121(017817)      645 LSR 647
CRN-like-2(017818)  543 LSR 545
```

Supplementary Figure S7. Scheme for *CRN* gene evolution.

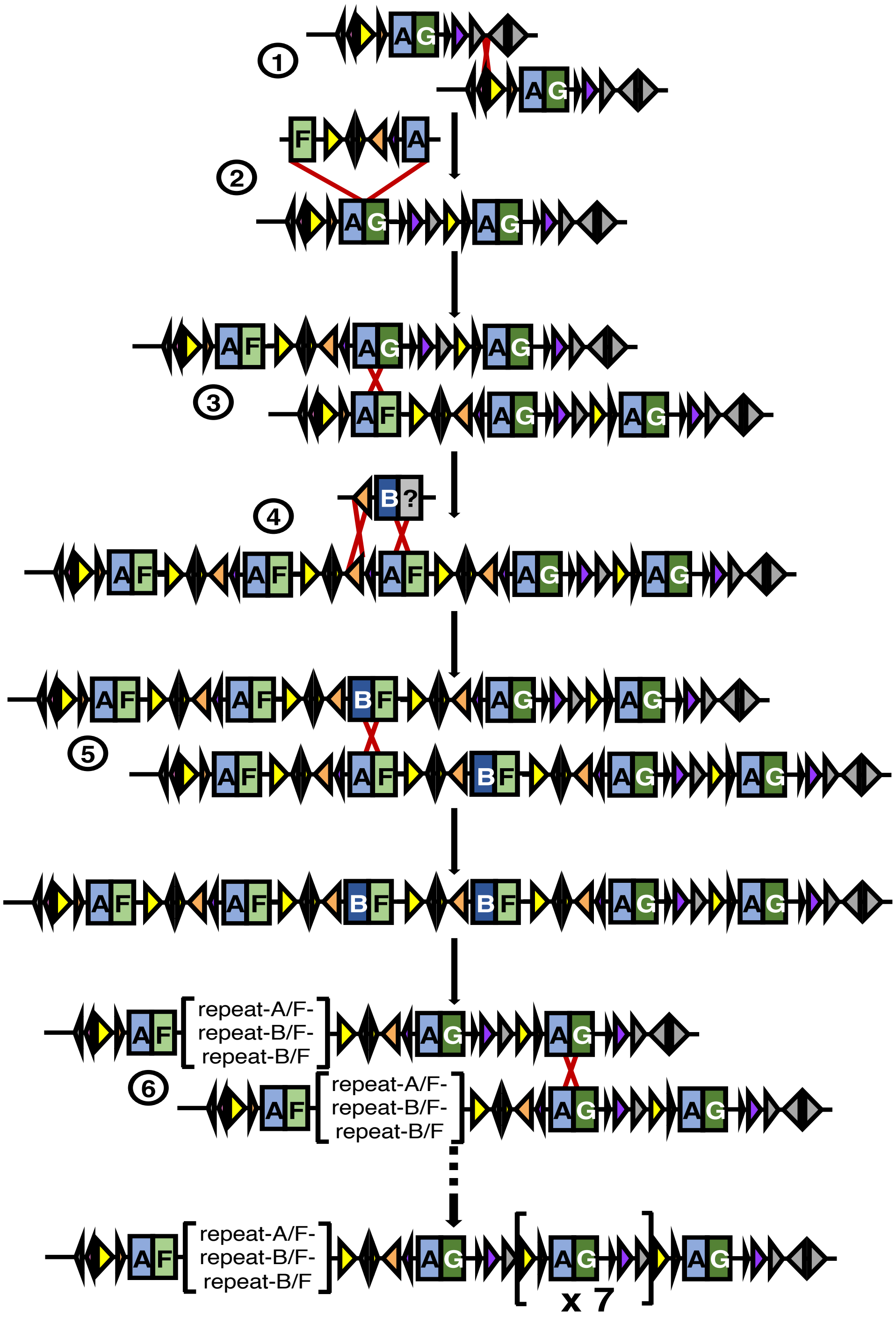

Potential sequence of events to generate the chromosome 2 *CRN* gene cluster in *Phytophthora agathidicida*. A sequence of recombination events, starting from a single *CRN* gene copy, that can explain the existing organization of the chromosome 2 *CRN* gene cluster is schematically diagrammed.

In step 1, an unequal recombination event results in duplication of an A/G *CRN* gene and gives a novel repeat block between the *CRN* copies. The repeat that mediated this recombination event is unclear: it may have been subsequently lost from the cluster or the event may have been microhomology-mediated. Step 2 involves an insertional recombination event, mediated by the recombination hotspot between the *CRN* gene N and C termini, that introduces a new *CRN* C-terminal variant and a new cluster of repeats to give an A/F *CRN* gene at the left edge. In step 3, two further unequal recombination events, each mediated by the recombination hotspot between the N and C termini of *CRN* genes, results in two A/F *CRN* gene duplications. Only one recombination event is depicted, but the result of both events is shown below. In step 4, a gene conversion event occurs between a *CRN* gene elsewhere in the genome and the right-most A/F *CRN* gene copy, mediated by the recombination hotspot between the N and C termini of the *CRN* genes and a transposon. This changes the A/F *CRN* gene into a B/F version and modifies the flanking repeat block. In step 5, an unequal recombination event mediated by the recombination hotspot between the N and C termini of the *CRN* genes occurs between the B/F *CRN* gene and the adjacent A/F copy, duplicating the B/F *CRN* gene. Finally, in step 6 a series of unequal recombination events mediated by the recombination hotspot between the N and C termini of the A/G *CRN* gene amplify A/G *CRN* gene copies. For ease of visualization, part of the cluster at this step is depicted in simplified text form between square brackets. A minimum of three unequal recombination events is required to generate the observed nine A/G *CRN* copies. Although shown as occurring last, the A/G *CRN* gene amplification could have occurred at any stage in this schema. Approximate recombination sites are indicated by red lines. Repeat and gene termini colors are as in Figure 7.

Supplementary Data 1

The *Phytophthora agathidicida* ribosomal RNA gene array (rDNA) locus.

The ribosomal RNA gene array (rDNA) was identified in the initial chromosome-level assembly at one location on chromosome 10. This was originally present as four complete identical rDNA copies, plus two partial flanking copies. However, inspection of the many rDNA repeat copies present in contigs generated during chromosome-level assembly revealed two common, different rDNA unit types that differ by just a single C indel at position 5,188; other mutations are not shared between more than two copies. Therefore, we amended the final assembly to only include two full repeats (for ease of mapping) plus the two partial flanking units, with one of the full units being the C deleted variant. The rDNA array (from the first partial flanking unit) starts at position 53,647 of chromosome 10, but rRNA is transcribed in the opposite direction. The canonical rDNA unit is 10,435 bp in length, with a 6,318 bp coding region and a 4,117 bp intergenic spacer region; sizes broadly consistent with those reported from oomycetes previously (Klassen, et al. 1987). Using a modal read counting method, we estimated the rDNA copy to be 245, which is somewhat lower than that measured previously (414) for *P. infestans* (Judelson and Randall 1998), but higher than is typical for the true fungi (Lofgren, et al. 2019). The high level of sequence identity found between rDNA copies in the assembly may indicate that the rDNA array is highly homogenized in *P. agathidicida* (Ganley and Kobayashi 2007), although it is possible the assembly has masked some intra-array variability.

References

Ganley ARD, Kobayashi T. (2007). Highly efficient concerted evolution in the ribosomal DNA repeats: total rDNA repeat variation revealed by whole-genome shotgun sequence data. Genome. Res. 17:184-191.

Judelson HS, Randall TA. (1998). Families of repeated DNA in the oomycete *Phytophthora infestans* and their distribution within the genus. Genome 41:605-615.

Klassen GR, McNabb SA, Dick MW. (1987). Comparison of physical maps of ribosomal DNA repeating units in *Pythium*, *Phytophthora* and *Apodachlya*. J. Gen. Microbiol. 133:2953-2959.

Lofgren LA, Uehling JK, Branco S, Bruns TD, Martin F, Kennedy PG. (2019). Genome-based estimates of fungal rDNA copy number variation across phylogenetic scales and ecological lifestyles. Mol. Ecol. 28:721-730.
